# Breath characteristics and adventitious lung sounds in healthy and asthmatic horses

**DOI:** 10.1111/jvim.16980

**Published:** 2024-01-08

**Authors:** Eloïse Greim, Jan Naef, Sophie Mainguy‐Seers, Jean‐Pierre Lavoie, Sophie Sage, Gaudenz Dolf, Vinzenz Gerber

**Affiliations:** ^1^ Swiss Institute of Equine Medicine (ISME), Department of Clinical Veterinary Medicine, Vetsuisse‐Faculty University of Bern Bern Switzerland; ^2^ Faculty of Veterinary Medicine, Department of Clinical Sciences University of Montréal St‐Hyacinthe QC Canada

**Keywords:** breath intensity, crackles, digital auscultation, equine asthma, rattles, wheeze

## Abstract

**Background:**

Standard thoracic auscultation suffers from limitations, and no systematic analysis of breath sounds in asthmatic horses exists.

**Objectives:**

First, characterize breath sounds in horses recorded using a novel digital auscultation device (DAD). Second, use DAD to compare breath variables and occurrence of adventitious sounds in healthy and asthmatic horses.

**Animals:**

Twelve healthy control horses (ctl), 12 horses with mild to moderate asthma (mEA), 10 horses with severe asthma (sEA) (5 in remission [sEA−], and 5 in exacerbation [sEA+]).

**Methods:**

Prospective multicenter case‐control study. Horses were categorized based on the horse owner‐assessed respiratory signs index. Each horse was digitally auscultated in 11 locations simultaneously for 1 hour. One‐hundred breaths per recording were randomly selected, blindly categorized, and statistically analyzed.

**Results:**

Digital auscultation allowed breath sound characterization and scoring in horses. Wheezes, crackles, rattles, and breath intensity were significantly more frequent, higher (*P* < .001, *P* < .01, *P* = .01, *P* < .01, respectively) in sEA+ (68.6%, 66.1%, 17.7%, 97.9%, respectively), but not in sEA− (0%, 0.7%, 1.3%, 5.6%) or mEA (0%, 1.0%, 2.4%, 1.7%) horses, compared to ctl (0%, 0.6%, 1.8%, −9.4%, respectively). Regression analysis suggested breath duration and intensity as explanatory variables for groups, wheezes for tracheal mucus score, and breath intensity and wheezes for the 23‐point weighted clinical score (WCS23).

**Conclusions and Clinical Importance.:**

The DAD permitted characterization and quantification of breath variables, which demonstrated increased adventitious sounds in sEA+. Analysis of a larger sample is needed to determine differences among ctl, mEA, and sEA− horses.

**Abbreviations**
3D: 3‐dimensional
ANOVA: analysis of variance
BAL: bronchoalveolar lavage
BALF: bronchoalveolar lavage fluid
CORSA: computerized respiratory sound analysis
ctl: healthy control horses
DAD: digital auscultation device
EA: equine asthma
exp: expiratory
HOARSI: horse owner‐assessed respiratory signs index
insp: inspiratory
IQR: interquartile range
mEA: mild to moderate equine asthma
PBS: phosphate‐buffered saline
PCM: pulse code modulation
R: resistance
SD: Secure Digital
sEA−: severe equine asthma in remission
sEA+: severe equine asthma in exacerbation
WCS23: 23‐point weighted clinical score
X: reactance

## 1 INTRODUCTION

Thoracic auscultation is a mainstay in the diagnostic evaluation of horses suffering from respiratory disorders,^1^, ^2^, ^3^ because it requires little equipment and is practical. In asthmatic horses, auscultation findings are part of several clinical scoring systems for asthma (EA) severity,^4^, ^5^, ^6^, ^7^ contributing 9 of 23 points to a widely adopted weighed clinical score (WCS23).^8^

However, routine auscultation of breath sounds suffers from 3 major limitations. First, short, dynamic, or narrowly localized auscultatory events may be missed.^9^, ^10^, ^11^ Second, correct classification of breath sounds is prone to biases and subjective.^12^, ^13^, ^14^ Third, no standardized and accepted nomenclature of respiratory sounds exists in veterinary medicine. Even in human medicine, despite the Computerized Respiratory Sound Analysis (CORSA) guidelines project,^3^ lung sound characterization is still a matter of debate.^15^, ^16^ Generally, adventitious sounds are divided into crackles and wheezes.^1^, ^2^, ^13^, ^17^ Wheezes are prolonged musical sounds caused by passage of air through narrowed airways. Crackles are short discontinuous sounds that can be further classified as fine or coarse. Fine crackles are believed to be generated by the reopening of collapsed airways, whereas coarse crackles, also known as rattles or mucus sounds heard over the trachea, are thought to be produced by passage of air through secretions.^15^, ^16^ Some horses with severe asthma (sEA) also can have quiet areas within the lung fields.^18^

Because of these limitations, recording and computer analysis of lung sounds in humans have received much attention in recent decades,^19^, ^20^, ^21^, ^22^, ^23^ culminating in the development of electronic stethoscopes supported by artificial intelligence for automatic diagnoses. In contrast, recording and digital analysis of lung sounds in horses, to our knowledge, has only been reported in a single case of pulmonary edema.^24^

Here, we introduce a wearable digital stethoscope designed to autonomously record auscultatory events over prolonged periods, in multiple locations simultaneously. Our first aim was to determine if this digital auscultation device (DAD) allowed characterization and quantification of breath variables (duration, variability, and intensity) and frequency of cough and adventitious sounds in healthy and asthmatic horses. Our second aim was to test the ability of breath sound quantification based on DAD recordings to discriminate auscultatory findings between healthy horses and those with varying degrees of asthma. We hypothesized that DAD would detect more adventitious sounds in asthma‐affected horses and that the frequency of these abnormal sounds would increase with severity of disease. We also expected associations of wheezing with exacerbation, of crackles with asthma severity overall, and of rattles with tracheal mucus accumulation.

## 2 MATERIALS AND METHODS

### 2.1 Study design and animal population

Our study was designed as an observational, analytical, prospective multicenter case‐control study conducted at the veterinary medical teaching hospital of the University of Bern (Switzerland) and at the Faculty of Veterinary Medicine of the University of Montréal (Canada).

Suitable candidates were identified using the horse owner‐assessed respiratory signs index (HOARSI).^25^ Briefly, horses were graded on a scale from 1 to 4 based on the current owner‐reported signs of respiratory disease (coughing, nasal discharge, breathing pattern, and performance). These grades represent unaffected (grade 1) to severely affected (grade 4) individuals. Horses with a HOARSI grade of 1 were included as controls (ctl), those with a grade of 2 or 3 entered the study as mild to moderate asthma (mEA) cases, and those with a grade of 4 as severe asthma (sEA) cases.

Based on data from a previous study with privately owned horses in Bern, power analysis indicated that 12 ctl and 12 mEA cases were needed to detect differences in adventitious lung sounds. Swiss client‐owned, adult horses presented to the ISME equine clinic as well as teaching horses of the University of Bern were enrolled as ctl or mEA during July to December 2022. From the research herd of the Faculty of Veterinary Medicine of the University of Montréal, 10 sEA horses (5 in exacerbation [sEA+] and 5 in remission [sEA−]) were enrolled, and data were collected in September 2022. These horses were diagnosed based on history (HOARSI 4) and previous results of lung function testing (transpulmonary pressure change >15 cm of H_2_O when stabled and fed hay) and bronchoalveolar lavage fluid (BALF) cytology (>25% neutrophils). To induce exacerbation, animals were stabled (wood shavings bedding) 4 weeks before the study and challenged by feeding hay. Management remained the same throughout the study period. No treatment was administered before collection of the data, except if horses showed inappetence, respiratory distress, or tachycardia (>60 beats per minute). Horses in remission were kept outside on pasture.

Clinical examination, thoracic ultrasound examination, airway endoscopy, BALF cytology and lung function (only in Montréal) were performed by the same clinician (EG). The results of these procedures were used to further characterize HOARSI‐assigned groups. In Bern, examinations were performed on the same day, once in each horse. In Montréal, all examinations (except thoracic ultrasound examination, which was not repeated after the first examination) were performed twice at intervals of 7 to 10 days. This approach resulted in 44 examination sessions and breath sound recordings (ie, once in 12 ctl and 12 mEA and twice each in 5 sEA− and 5 sEA+).

### 2.2 Clinical examination

Horses were weighed and complete physical examinations including attitude, appetite, heart rate, body temperature, and mandibular lymph node palpation were performed. The respiratory system was specifically examined using the WCS23^8^ during spontaneous breathing. Subsequently, ultrasound examination, digital auscultation and recording, lung function measurements, endoscopic examination and BALF collection were performed in that order, except when otherwise stated.

### 2.3 Thoracic ultrasound examination

A thoracic ultrasound examination was carried out on all the horses to delimit the respiratory field, measure skin—pleural distance, detect signs of infectious disease, and detect fields potentially interfering with auscultatory findings (eg, scars, masses, free fluid). The examination was performed starting from the 17th to the 3rd intercostal space, and from dorsal to ventral on both sides (Bern: SonoSite Titan, Bothell, WA; Montréal: ExaPad, IMV Imaging, Bellshill, Scotland).

### 2.4 Digital auscultation device

Respiratory sounds were obtained from 3‐dimensional (3D)‐printed stethoscope heads using microphones with extended low‐frequency response. Stethoscope heads were spring‐suspended in 3D‐printed enclosures pressed to the horse with elastic belts. This approach eliminated friction noise from the belts and standardized the contact pressure of the stethoscope membrane. Outside noise was further decreased by electronic noise canceling. Breath sounds were amplified, low pass filtered, and digitized within the recording heads with 16 bits per sample at a frame rate of 4410 Hz. Thoracic excursions during inhalation and exhalation were recorded by an inductive plethysmography belt located at the 15th intercostal space and digitized similarly. Samples from all recording heads and the plethysmography unit were transmitted to a central unit and saved on a Secure Digital (SD)‐card in a multitrack pulse code modulation (PCM) WAV file. Subjective comparisons using a commercial recording stethoscope suggested the superior sound quality of the novel device.

### 2.5 Collection of respiratory sound data

**FIGURE 1:**
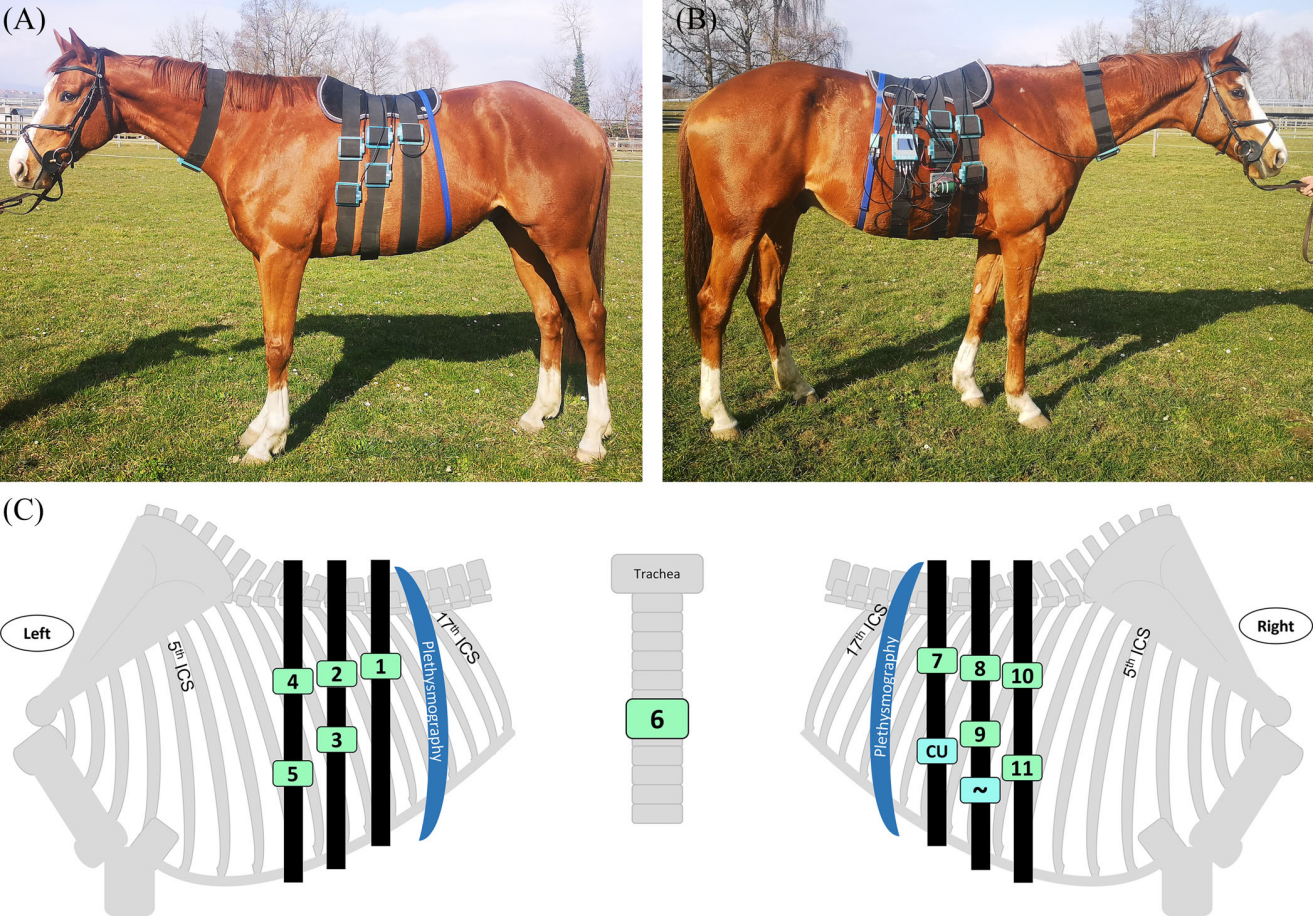
Photos (A and B) and illustration (C) of the digital auscultation device on a horse. The device included caudodorsal sensors 1 and 7, which were positioned on the 13th intercostal space (ICS). The middle elastic belt held sensors 2 and 8 on the 11th intercostal space, while sensors 3 and 9 were placed on the 10th intercostal space. The right side of the device held the battery beneath sensor 9, supplying power to the central unit (CU), which was located below sensor 7. The cranial belt secured sensors 4 and 10 on the 9th intercostal space and sensors 5 and 11 on the 8th intercostal space, respectively. Sensor 6 was attached ventral to the trachea. The plethysmography was placed around the horse's abdomen in the caudal region of sensors 1 and 7.

For each recording session, 1 sensor was placed over the trachea, and 5 sensors were placed on each side of the thorax and held in place by 3 elastic straps (Figure 1). The plethysmography belt was placed caudally, behind the last elastic strap fixing the 2 caudodorsal sensors at the 15th intercostal space. To avoid pressure sores, a padded mat was placed on the horse's back. Cables connected each sensor to the central unit (placed on the right side, below the caudodorsal sensor) and the latter was powered by a battery attached to the right side of the 2nd ventral elastic belt. The gain was standardized for all recordings and the sound quality was evaluated using headphones before starting the recording. We recorded for 60 minutes in a quiet stable away from other horses and without sedation. The data recorded on the SD card then were transferred to a computer to visually assess the quality of the recording on spectrograms (Audacity version 3.1.1 for Windows).

### 2.6 Analysis and classification of the respiratory lung sounds

**FIGURE 2:**
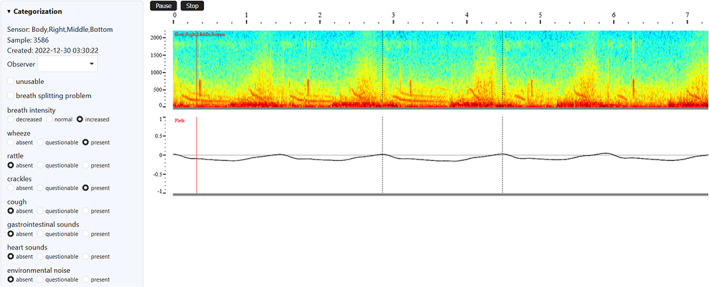
Selected breaths presented to the observer, along with two breaths each before and after for context. Sensor location, spectrogram of the breath sound, and waveform of the plethysmography data could be visually assessed, and the sound could be heard through headphones.

One‐hundred random breathing cycles per recording were randomly selected for blind analysis by the same observer (EG). This sample was weighted with 20% for the tracheal sensor and the remaining 80% equally divided for each lung sensor (8% per sensor). A total of 4400 breaths from the 44 recording sessions were presented to the observer in random order using a web application, along with an input mask for their classification (Figure 2). For contextual reference, the observer visualized 2 adjacent breaths before and after the breath to be classified. Additional information, such as sensor location, a spectrogram of the breath sound, and the waveform of the plethysmography data, were provided. Simultaneously, the sound could be heard through headphones with good low‐frequency capability. Wheeze, rattle (coarse crackle), crackle (fine crackle), cough, gastrointestinal sounds, heart sounds and environmental noise were assessed as absent, questionable, or present, and breath intensity as decreased, normal or increased. Multiple attributes could be selected for the same breath. Before the start of the analyses, the primary observer (EG) performed training classification of 800 breaths, of which 200 also were classified independently by a second observer (VG). Interobserver discrepancies were discussed until agreement was reached to minimize the effect of observer experience, presumably most pronounced at the beginning of the categorization phase.

### 2.7 Lung function tests

Lung function tests of sEA horses in Montréal were performed using the Equine MasterScreen impulse oscillometry system (Jaeger GmbH, Würzburg, Germany) in unsedated horses standing in stocks as previously described.^26^, ^27^ On each test day, the system was calibrated. Briefly, an airtight facemask was placed on the horse's head allowing multifrequency impulses produced by a loudspeaker to be superimposed to the tidal breathing of the horse with simultaneous acquisition of the pressure‐flow signal response of the respiratory system by pressure transducers connected to a pneumotachograph. Three consecutive 30‐second recordings were completed for each horse, except for 1 horse where recordings could not be made because of intolerance to the examination. Lung function data were acquired using LabManager (version 4.53, Jaeger, Würzburg, Germany) and analyzed with FAMOS (IMC, Meβsysteme, Berlin, Germany) using the fast Fourier transform method. Inspiratory (insp), expiratory (exp) and whole breath resistance (R), reactance (X), and coherence of respiratory system at frequencies of 5 and 10 Hz were analyzed. For the first assessment of 3 horses, the lung function was performed 1 day after the other examinations because of technical problems.

### 2.8 Endoscopic tracheal mucus scoring and BALF cytology

Horses were sedated with either xylazine (Montréal) (Nerfasin 100, Dechra, Pointe‐Claire, QC, Canada; 0.5 mg/kg, IV) or detomidine (Bern) (Equisedan, Dr. E. Graeub AG, Bern, Switzerland; 10 μg/kg, IV) and butorphanol (Montréal: Dolorex, Merck & Co, Inc, Rahway, NJ; 20‐30 μg/kg, IV; in Bern: Morphasol, Graeub AG, Bern, Switzerland; 10 μg/kg, IV). Tracheal mucus accumulation was scored along the entire length of the trachea using endoscopy (Bern: VET‐OR1200HD, Medical Solution GMBH, Wil, Switzerland; Montréal: Evis Exera II CV‐180, Olympus Canada Inc, Richmond Hill, ON, Canada) using a validated scoring system.^28^

Bronchoalveolar lavage fluid (BALF) collection and cytology methods slightly differed between Bern and Montréal. In Montréal, bronchoalveolar lavage (BAL) was performed under endoscopic guidance as previously described.^29^ Briefly, the endoscope was advanced down into the right lung until its tip was wedged in the wall of a bronchus. After topical anesthesia with 0.5% lidocaine (Lurocaine; lidocaine hydrochloride 20 mg/mL, Vétoquinol N.‐A. Inc, Lavaltrie, QC, Canada), two 250‐mL boluses of warm sterile isotonic saline then were sequentially instilled into the bronchus through the endoscope's biopsy channel and aspirated by a suction pump (10‐20 mmHg). The samples were kept on ice until reaching the laboratory, where cytocentrifuged preparations of BALF (400 μL) were made and cells were stained with a modified Wright‐Giemsa solution (DiffQuick, Fisher Scientific, Waltham, MA). A blinded investigator (SMS) performed differential leukocyte counts using 400 cells. In Bern, as previously described,^30^ a sterile BALF tube (either 240 or 300 cm long depending on horse size; 10 mm outer diameter; Bivona Medical Technologies) was used for BALF collection. Briefly, the catheter was passed through the nostrils and trachea until it was wedged in a bronchus. Lidocaine (Lidocain 2% Streuli ad us. vet., Uznach, Switzerland) was administered, followed by instillation of 0.9% NaCl solution. The volume of lidocaine and NaCl used depended on the horse's weight, with <300 kg horses receiving 15 mL lidocaine and 200 mL NaCl, whereas horses weighing ≥300 kg received 20 mL of lidocaine and 250 mL of NaCl. The solutions were gently withdrawn manually using 60‐mL syringes. The BALF was pooled in a 250‐mL silicone‐coated glass bottle (Fisher Scientific, Leicestershire, UK), and the amount of liquid collected was recorded. The BALF was filtered through a 40 μm cell strainer (Falcon, FisherScientific, Leicestershire, UK) and sent to the laboratory, where it was first centrifuged for 3 minutes at 500*g*. A volume of 100 μL of the sediment was combined with an additional 100 μL of phosphate‐buffered saline (PBS). The solution was cytocentrifuged a second time for 8 minutes at 1000*g*. Finally, slides were automatically stained with Wright‐Giemsa and differential leukocyte counts of 200 cells were performed by laboratory technicians.

### 2.9 Statistical analysis

Statistical analyses were done using R version 4.3.1 (R Core Team 2023).^31^ Group differences in means of age, weight, body condition score, skin—pleural distance, WCS23, tracheal mucus score and BALF cytology were assessed by Welch's analysis of variance (ANOVA) and Games‐Howell post hoc tests for pairwise comparisons. Differences in corresponding medians were evaluated using heteroscedastic one‐way ANOVA.

Except for the repeated measurements in the sEA cases, all statistical analyses were performed using only the first examination sessions, resulting in 34 complete examination sessions (3400 breaths).

The proportions of analyzable breaths that contained adventitious sounds (wheezes, crackles, and rattles) and the respiratory variables (breath intensity, mean and variance of breath duration) were compared across groups (ctl, mEA, sEA−, sEA+) using Kruskal‐Wallis tests and Dunn tests for pairwise comparisons, with P‐values adjusted with the Holm method. Coughing episodes from the 3400 samples did not present sufficient events for statistical analysis. Breath intensity was calculated as the difference between the proportions of increased and decreased breath sounds, leading to a value between −100% and +100%. The proportion of adventitious sounds was calculated by adding the proportions of sounds labeled as “present” with weight 1 and those labeled as “questionable” with weight 0.5. Mean and variance of breath duration were calculated from the plethysmography data of the entire recordings.

The differences between repeated measurements in the sEA cases (means and variance of respiratory duration, breath intensity, ratio of impulse oscillometry system resistance at 5 and 10 Hz, impulse oscillometry system reactance at 5 Hz, proportion of wheezes, crackles, and rattles, tracheal mucus score, neutrophilic percentage of BALF cytology and WCS23) were assessed using 2‐sided 1‐sample *t*‐tests. Multiple comparisons were accounted for using Bonferroni correction.

The predictive value of auscultation findings was assessed using general linear models, with group, neutrophilic percentage on BALF cytology, tracheal mucus score, and WCS23 as response variables and breath characteristics (breath duration and variability, breath intensity, wheezes, crackles, rattles, cough) as predictors, controlling for skin‐pleural distance, age, and sex. For these models, the proportions of abnormal sounds (wheezes, crackles, rattles, and coughs) were transformed to new variables with 3 levels (abnormal sound absent; at least 1 breath with questionable abnormal sound; at least 1 breath with present abnormal sound). Model selection was based on the Bayesian information criterion. A difference of 2 to 6 in the Bayesian information criterion is needed for positive evidence in favor of a specific model.^32^

## 3 RESULTS

### 3.1 Horse population characteristics

Thirty‐four horses were included in the study, 11 geldings and 23 mares between 5 and 32 years of different breeds: 12 American Quarter Horses, 9 Warmbloods, 5 Freibergers, 3 Thoroughbreds, 2 Trotters, 5 Paint Horses, 2 Canadians, 2 Grade horses, 1 Arabian, 1 Camargue, 1 Haflinger, and 1 Italian pony. All horses were in good body condition and had normal appetite and attitude, and normal body temperature and heart rate. No significant differences in age or sex were found among the groups, but WCS23, tracheal mucus score, and percentage of neutrophils, lymphocytes, and mast cells in BALF cytology differed significantly between groups (*P* < .05, Table 1). Comet‐tail artifacts were seen on thoracic ultrasound examination in most horses regardless of clinical status (as previously described^33^), but no other abnormalities such as consolidations or free fluid were observed.

**TABLE 1:** Characteristics of the study sample of horses.

	Ctl	mEA	sEA−	sEA+	
	(n = 12)	(n = 12)	(n = 5)	(n = 5)	
	Median [Range]	Median [Range]	Median [Range]	Median [Range]	*P*‐value
Age (years)	13.5 [6‐32]	11.5 [5‐19]	18.0 [17‐22]	17.0 [15‐22]	.07
Weight (kg)	575.5 [513‐665]	539.0 [258‐618]	498.0 [415‐602]	513.0 [498‐626]	.14
BCS (1‐9)	6.0 [5‐9]	6.0 [5‐8]	6.0 [4‐7]	6.0 [4‐9]	1.00
Skin‐pleura (mm)	36.5 [23.0‐60.0]	36.6 [23.0‐54.0]	34.2 [19.2‐44.1]	29.0 [20.8‐51.5]	.78
WCS23	1.5 [0‐5]	5.5 [2‐16]	3.0 [2‐4]	17.0 [14‐19]	<.01
TMA (1‐5)	0.5 [0.0‐2.0]	3.0 [0.0‐5.0]	2.0 [1.0‐3.0]	3.0 [1.0‐3.5]	<.01
BAL mac (%)	48.3 [29.5‐74.0]	27.5 [5.5‐76.0]	33.8 [24.8‐58.3]	26.3 [15.8‐31.3]	.10
BAL lym (%)	38.5 [20.5‐60.0]	14.0 [6.0‐57.5]	45.3 [39.0‐69.5]	51.3 [40.0‐64.3]	<.01
BAL neu (%)	5.5 [2.0‐31.5]	42.0 [12.0‐78.0]	5.5 [1.8‐29.8]	22.3 [5.0‐43.3]	.03
BAL mast (%)	2.3 [0.0‐7.5]	2.3 [1.5‐7.0]	0.3 [0.0‐0.8]	1.0 [0.3‐2.0]	<.01
BAL eos (%)	0.0 [0.0‐1.5]	0.5 [0.0‐17.0]	0.0 [0.0‐0.0]	0.0 [0.0‐0.0]	.42

Abbreviations: BAL eos, BAL eosinophils; BAL lym, BAL lymphocytes; BAL mac, BAL macrophages; BAL mast, BAL mastocytes; BAL neu, BAL neutrophils; BCS, body condition score; Ctl, healthy control horses; mEA, mild to moderate equine asthma; sEA+, severe equine asthma in exacerbation; sEA−, severe equine asthma in remission; TMA, tracheal mucus accumulation; WCS23, 23‐point‐weighted‐clinical score.

In accordance with the inclusion criteria, all ctl horses had a HOARSI of 1, and all cases had a HOARSI ≥2 (7 mEA with HOARSI = 2, 5 mEA with HOARSI = 3, 10 sEA with HOARSI = 4). In sEA horses in Montréal, exacerbation was confirmed during lung function assessment based on the ratio of resistance at 5 and 10 Hz being ≥1 and the reactance at 5 Hz being ≤0. Of the 5 sEA+ horses, 1 mare did not tolerate the examination and required inhalation with 1000 μg of Salbutamol (Ventolin, GSK, Mississauga, ON, Canada) at both examinations because of respiratory distress. The status of all sEA− horses was further confirmed using lung function measurements with the ratio of resistance at 5 and 10 Hz being ≤1 and the reactance at 5 Hz being ≥0.^26^

### 3.2 Quality assessment of the digital auscultation device and sound characterization

Each horse underwent simultaneous auscultation at 11 locations for 1 hour, resulting in a high number of breath recordings per horse, ranging from 5478 to 27 852. Of all recorded breaths, 96.3% were correctly identified from the plethysmography data and 85.9% were classified as usable, enabling characterization and categorization according to the predetermined criteria. Among nonrespiratory sounds, environmental noises were present in 30.9% and questionably present in 2.3% of the usable samples. Most of the environmental noise was caused by external air ventilation or road traffic. Gastrointestinal sounds also were present in 28.8% and questionably present in 11.1% of the samples. Heart sounds were noted less frequently, with presence at 1.9% and questionable presence at 0.8%.

Training classification of 800 breaths and comparisons with the second observer permitted characterization of the respiratory sounds before the start of scoring. Coughs were characterized by a wide vertical band with high intensity and a broad frequency spectrum >2000 Hz. Crackles and rattles consisted of sequences characterized by high‐frequency and high‐intensity peaks, occasionally presenting challenges in differentiation because of the similarities. Crackles tended to be present in the inspiratory phase, whereas rattles were present in both the inspiratory and expiratory phases. When heard through headphones, the sound of crackles was similar to Velcro opening, whereas rattles were reminiscent of a snoring sound. Both sounds also could resemble the rubbing of the stethoscope against the skin, leading to potential confusion. These similarities and artifacts led to a higher proportion of these sounds being categorized as questionable or potentially as false positive. Indeed, in the final sample, the proportions of questionable crackles (overall scorings: 5.4% questionable, 8.1% present) and rattles (3.2% questionable, 4.3% present) were considerably higher than of wheezes (1.9% questionable, 9.9% present).

Wheezes appeared as thin transverse bands between 200 and 1000 Hz, providing the most distinctive signal both audibly and visually. Most questionable wheezes were likely a result of potential confusion with intestinal sounds transmitted from the abdomen into the thoracic cavity. The high frequency of gastrointestinal sounds, which were noted in more than 33% of the scored breaths overall, necessitated a strategy to improve distinction. The training phase showed that wheezes typically occurred at the end of expiration, as previously described,^34^ whereas gastrointestinal sounds were not associated with the respiratory phase. Here, parallel visual inspection of the spectrogram and plethysmography curve was particularly helpful. Additionally, wheezes often occurred in series (commonly present in the breaths preceding and following the analyzed breath), facilitating differentiation from gastrointestinal noises.

Examples of typical adventitious breath sounds can be found as visual representations and as audio files in Supplementary Item 1.

### 3.3 Respiratory variables and adventitious sounds in healthy and asthmatic horses

**FIGURE 3:**
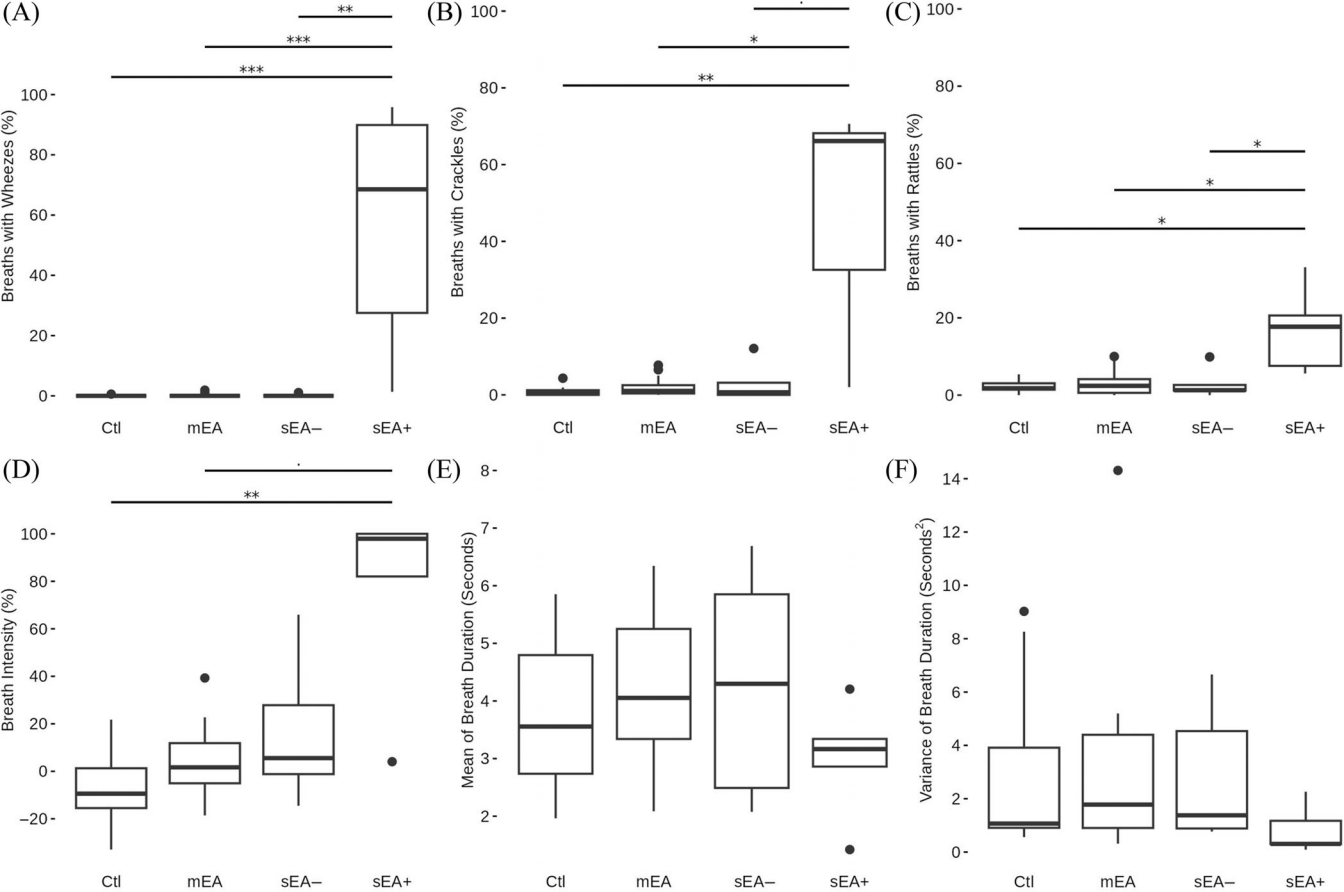
Percentage of the adventitious sounds (wheezes, A; crackles, B; rattles, C), breath intensity (D), as well as mean (E) and variance (F) of breath duration for each group (ctl, mEA, sEA−, sEA+). ^•^ *P* < .1; *P < .05; **P < .01; ***P < .001.

Groups differed significantly in the proportion of breaths with wheezes (*P* < .001), rattles (*P* = .01) and crackles (*P* < .01) and in breath intensity (*P* < .01), whereas mean or variance of breath duration were not significantly different among groups (Figure 3). Pairwise comparisons showed that these variables were consistently higher in sEA+ compared to all other groups. This was particularly marked for wheezes where sEA+ had a median at 68.6% (interquartile range [IQR], 27.5%‐89.9%) whereas ctl, mEA and sEA− were at 0% (0%‐0% each). Crackles and rattles followed the same pattern, with medians for sEA+ at 66.1% (IQR, 32.6%‐68.2%) and 17.7% (IQR, 7.6%‐20.6%), respectively, and medians for ctl, mEA and sEA− at 0.6% (IQR, 0%‐1.2%) and 1.8% (IQR, 1.4%‐3.0%), 1.0% (IQR, 0.4%‐2.5%) and 2.4% (IQR, 0.6%‐4.1%), and 0.7% (IQR, 0%‐3.2%) and 1.3% (IQR, 1.2%‐2.6%), respectively. The medians for respiratory intensity showed a progressive increase to −9.4% (IQR, −15.5% to 1.3%) for ctl, 1.7% (IQR, −5.0% to 11.9%) for mEA, 5.6% (IQR, −1.2% to 27.8%) for sEA− and a much higher value of 97.9% (IQR, 82.0%‐100%) for sEA+, with significant differences between sEA+ and ctl.

### 3.4 Measurement repeatability in sEA cases

The differences between repeated measures of mean and variance of breath duration, breath intensity, lung function scores (ratio of impulse oscillometry system resistance at 5 and 10 Hz, impulse oscillometry system reactance at 5 Hz), proportions of adventitious sounds (wheezes, crackles, rattles), tracheal mucus score, and neutrophilic percentage on BALF cytology, and WCS23 were not significantly different from zero (all *P* = 1; except for variance of breath duration, *P* = .63), indicating stable clinical status for sEA− and sEA+. Statistical results and a graphical representation of this analysis can be found in Supplementary Item 2.

### 3.5 Linear regression analyses

Mean breath duration and breath intensity were positively associated with asthma severity (estimate = 0.53, *P* = .07; estimate = 0.07, *P* < .01, respectively).

We found no significant association of the tested predictors with neutrophil percentage on BALF cytology.

Wheeze was positively associated with tracheal mucus score (estimate = 0.63, *P* = .03). Wheeze explained 14% of the variance of the tracheal mucus score.

Breath intensity and wheeze were positively associated with WCS23 (estimate = 0.08, *P* < .01; estimate = 2.40, *P* = .06, respectively).

## 4 DISCUSSION

According to our first aim, the novel DAD provided high‐quality recordings over an extended period and over multiple lung fields simultaneously. It allowed characterization and quantification of breath duration and variability, breath intensity as well as adventitious sounds described in conventional auscultation of asthmatic horses.

All adventitious sounds recorded could be characterized using the predefined categories. Typical examples of wheezes, crackles and rattles agreed with clinical descriptions and sound analyses of auscultatory recordings in horses and humans.^7^, ^15^, ^18^, ^34^, ^35^ Nevertheless, categorization of a considerable proportion of adventitious sounds remained equivocal, with the highest confidence attained for wheezes, followed by crackles and rattles. No other adventitious sounds, such as pleural rubs, were detected, which was expected because horses had no evidence of pleuritis on ultrasonographic screening. Coughing could be clearly identified on digital recordings, but the numbers of observations were too low for meaningful statistical analyses.

Once the first objective was achieved, the investigation progressed toward the second goal, which aimed to assess the ability of DAD to distinguish healthy horses from those with variable degrees of asthma. Compared to clinically healthy controls, all adventitious sounds were more frequent in sEA+, but not in sEA− and in mEA. Wheezing was almost exclusively heard in sEA+. Among the other groups, this adventitious sound only was identified at very low proportions of <2% of breaths in a few individuals. In contrast, sEA+ often displayed remarkably frequent wheezes, which were heard in >50% of the scored breaths in this extreme phenotype. Differences were less marked for crackles and rattles, which were heard at low proportions of 7% to 12% of scored breaths in some mEA or sEA− (individual results not shown). In the small number of severely affected horses examined, occurrence of adventitious sounds appeared remarkably constant at repeated examinations, both in remission and in exacerbation.

Although mEA and sEA− seemed to have louder breath sounds than ctl, these differences were not significant. Also, mean breath duration and variance of breath duration did not differ among groups. This finding contrasted with our expectations, because asthmatic horses are known to exhibit more regular and rapid breathing.^36^ Otherwise, our findings agree with reports of abnormal lung auscultation findings that were unmistakably present in severe,^6^, ^18^ but absent in milder forms of EA, apart from increased breath intensity and occasional subtle wheezes.^7^, ^37^

In our study, increased breath sound intensity and duration were associated with the study groups. Breath intensity and presence of wheezes were explanatory variables for the WCS23. Auscultation findings are an important part of the WCS23,^8^ which could have biased this association. Wheezing also was associated with tracheal mucus accumulation. We had expected that crackles and particularly rattles would be heard when excessive airway secretions are present, as described in human medicine,^15^ but no such association was found in our data. Adventitious sounds and the other respiratory variables examined also were not predictive of the degree of BALF neutrophilia.

Because of the limited number of observations, we combined the tracheal and thoracic recordings for statistical analyses. Air movement during breathing is fastest in the large airways and thus best heard over the trachea. Therefore, tracheal sounds might be different in character from those heard over the thorax. Separate analysis of tracheal sounds in future investigations could improve sensitivity, particularly for rattles. Furthermore, we did not conduct a separate analysis of the signals captured by the thoracic stethoscopes, which potentially could enhance the sensitivity in detecting wheezes and crackles. This consideration is based on clinical experience and lung physiology, because these abnormal sounds are believed to be more effectively detected in the caudodorsal lung fields. Separate spatio‐temporal analyses of the thoracic sensor signals also might identify areas of relatively decreased breath intensity (silent fields). Similarly, results from the digital evaluation could not be directly compared to the clinical scoring of breath sounds used in the WCS23, because adventitious sound definitions were not identical and also would necessitate separate evaluation of tracheal and thoracic sensor recordings. In our study, these analyses were not possible, because breath samples from tracheal and all thoracic sensors were pooled and then assessed in a randomized fashion.

Compared to routine thoracic auscultation performed in horses, we were able to evaluate a larger number of breaths in a concentrated and standardized fashion by the same blinded observer, who further profited from visual aids complementing the auditory signals. In lung sound recordings from normal and diseased humans, spectrograms improved the classification of wheezes and crackles.^35^ In our study, the synchronized presentation of spectrograms and plethysmography curves with auditory signals was deemed very helpful to categorize adventitious sounds and differentiate them from direct mechanical, external environmental, and gastrointestinal sounds.

Although the challenges posed by the inherent subjectivity of lung sound assessment were mitigated by standardized signal acquisition and blinded breath scoring, a limitation of our study was the combination of small sample size and imperfect group definitions. Clinical status was used for recruitment and group assignment, and standard diagnostic methods, including the WCS23, endoscopic scoring of tracheal mucus accumulation, BALF cytology, and lung function were employed for characterization of the study population. The BALF cytology, considered the gold standard for differentiation of ctl and mEA, showed that a substantial proportion of ctl could not be considered normal according to strict consensus standards.^37^ Also, a higher median percentage of neutrophils was recorded in the mEA group compared to sEA+. The latter may have included individuals with paucigranulocytic asthma or falsely low neutrophil percentages in samples with decreased BALF return. Overall, the small sample sizes per group likely resulted in more pronounced effects of extreme values.

Nevertheless, we could show that adventitious sounds are a consistent finding only in sEA+, a phenotype that does not pose substantial diagnostic challenges because of the obvious dyspnea and frequent coughing easily appreciated by clinicians and owners. In contrast, milder forms of asthma often necessitate more invasive diagnostic examinations such as BALF cytology. Our results do not allow us to estimate the value of auscultation to detect adventitious sounds in mEA. Even with digital auscultation, differences between mEA and ctl were not significant, and routine auscultation is likely less sensitive. Transitory pathological breath sounds easily may be missed during brief routine examinations or, alternatively, they may be overinterpreted because of expectation bias. Interestingly, we found that the arguably most subjective measure, breath intensity, could hold the most promise to differentiate between ctl and mEA.

Although lung auscultation is considered a cornerstone of respiratory tract examination, our study represents the first time that auscultatory findings were investigated systematically in asthmatic horses. In human medicine, the quantification of wheezing in nocturnal asthma has been used to assess the severity of the disease and monitor response to treatment.^38^, ^39^ Because of the time‐consuming procedure employed for blinded assessment by the same observer, we only analyzed a small proportion of the total recorded breaths (<1%). In the future, machine learning algorithms for breath sound recognition could facilitate the scoring of a larger number of good quality auscultatory recordings to further investigate the value of standardized and automated analyses of lung auscultation. This approach could enable investigation of spatio‐temporal patterns of lung sounds across the 5 locations on each side of the thorax. Furthermore, automated analyses of spontaneous respiration and rebreathing examinations could be compared with unblinded routine auscultation findings.

In conclusion, the novel DAD permitted assessment of respiratory variables and quantification of adventitious lung sounds in healthy and asthmatic horses. It also permitted distinction between ctl and sEA+. In sEA+, all adventitious lung sounds were markedly increased. By overcoming some of the limitations of traditional auscultation methods, this digital approach can provide more accurate and objective assessments of respiratory conditions in horses, potentially supporting diagnosis, monitoring, and management of EA. However, additional analyses on larger samples of well‐characterized horses are required to determine its ability to discriminate mEA from ctl. Also, further research in this field could explore the application of digital auscultation in other respiratory disorders in clinical practice.

## CONFLICT OF INTEREST DECLARATION

Authors declare no conflict of interest.

## OFF‐LABEL ANTIMICROBIAL DECLARATION

Authors declare no off‐label use of antimicrobials.

## INSTITUTIONAL ANIMAL CARE AND USE COMMITTEE (IACUC) OR OTHER APPROVAL DECLARATION

Approved by the university and cantonal Animal Experimentation Committees (VD3624b, BE4/20+, and BE89/2022) and the committee for animal use of the University of Montréal (Protocol # 22‐Rech‐2202).

## HUMAN ETHICS APPROVAL DECLARATION

Authors declare human ethics approval was not needed for this study.

## Supporting information


**Data S1. Supplementary Item 1.** Visual representation of breath sounds as waveform (top) and spectrogram (middle), and plethysmography breathing pattern (bottom) where increasing values represent inspiration and decreasing values expiration. The x‐axis represents time in seconds. In the waveform, the y‐axis represents the amplitude or “loudness.” In the spectrogram, the y‐axis represents frequency in Hertz, and the intensity of sounds is represented as nuance color. (A) Four normal respiratory cycles over the lungs, (B) 6 normal respiratory cycles over the trachea, (C) 5 respiratory cycles over the lungs containing expiratory wheezes (thin transverse bands at 700 Hz), (D) 14 respiratory cycles over the lungs containing both wheezes (500 Hz) and crackles (high‐frequency and high‐intensity peaks), (E) 4 respiratory cycles over the lungs of increased intensity containing crackles, (F) 5 respiratory cycles over the trachea containing rattles (high‐frequency and high‐intensity peaks) and expiratory wheezes, (G) series of 3 coughs over the lungs containing wheezes, as well as an isolated wheeze between the second and third cough. The audio files corresponding to each figure are appended in the supplementary items.

**Data S2. Supplementary Item 2.** Supplementary figure and table.
