# Supplementary figures and images for: Breath characteristics and adventitious lung sounds in healthy and asthmatic horses
Source: J Vet Intern Med. 2024 Jan 8;38(1):495–504. doi: 10.1111/jvim.16980 (PMC10800186; doi:10.1111/jvim.16980)

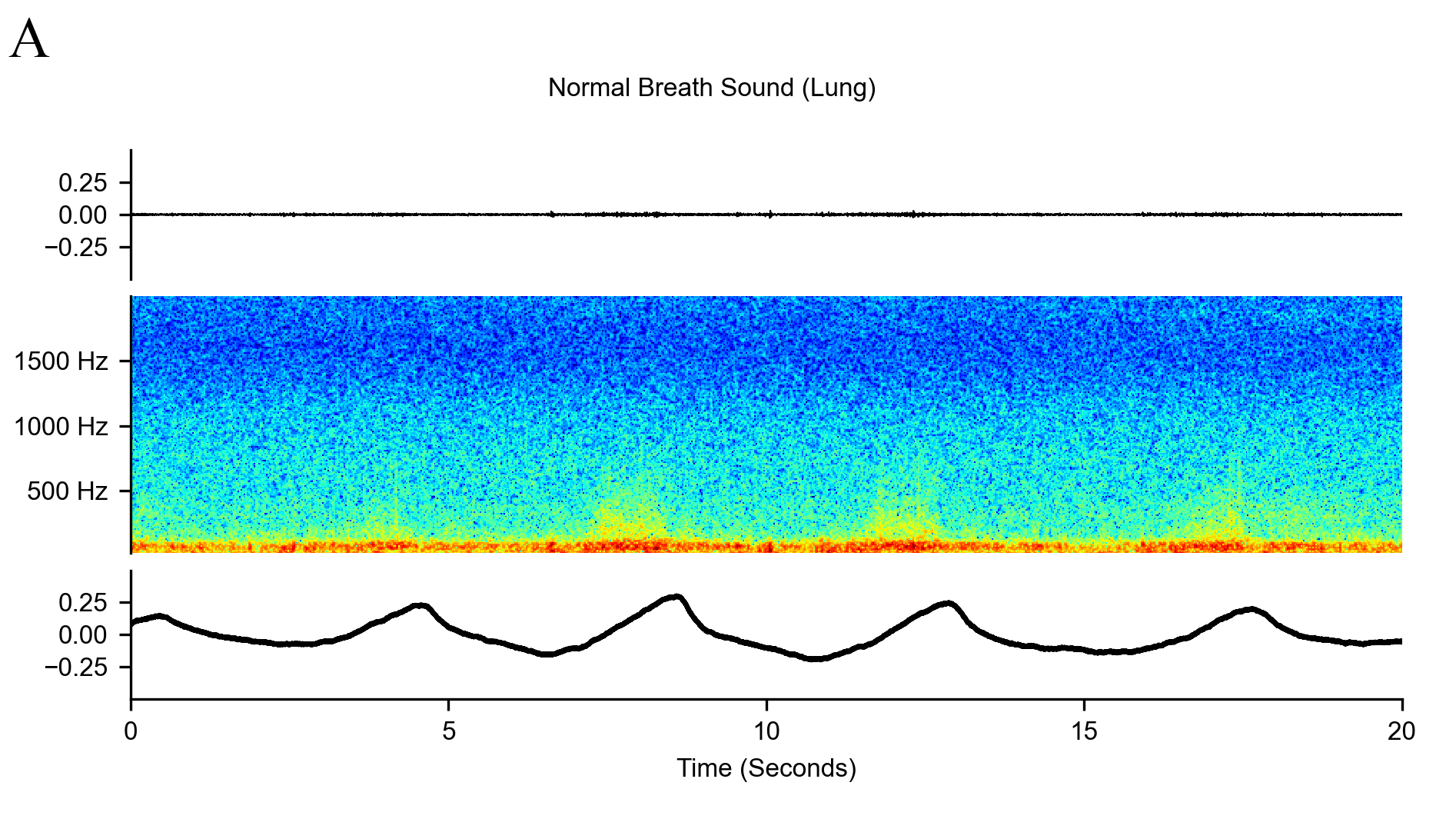

Supplement: Supplementary file 1 — Data S1. Supplementary Item 1. Visual representation of breath sounds as waveform (top) and spectrogram (middle), and plethysmography breathing pattern (bottom) where increasing values represent inspiration and decreasing values expiration. The x‐axis represents time in seconds. In the waveform, the y‐axis represents the amplitude or “loudness.” In the spectrogram, the y‐axis represents frequency in Hertz, and the intensity of sounds is represented as nuance color. (A) Four normal respiratory cycles over the lungs, (B) 6 normal respiratory cycles over the trachea, (C) 5 respiratory cycles over the lungs containing expiratory wheezes (thin transverse bands at 700 Hz), (D) 14 respiratory cycles over the lungs containing both wheezes (500 Hz) and crackles (high‐frequency and high‐intensity peaks), (E) 4 respiratory cycles over the lungs of increased intensity containing crackles, (F) 5 respiratory cycles over the trachea containing rattles (high‐frequency and high‐intensity peaks) and expiratory wheezes, (G) series of 3 coughs over the lungs containing wheezes, as well as an isolated wheeze between the second and third cough. The audio files corresponding to each figure are appended in the supplementary items. [file JVIM-38-495-s002.zip › Supplemetary figure 1A_Normal Breath Sound (Lung).tif]

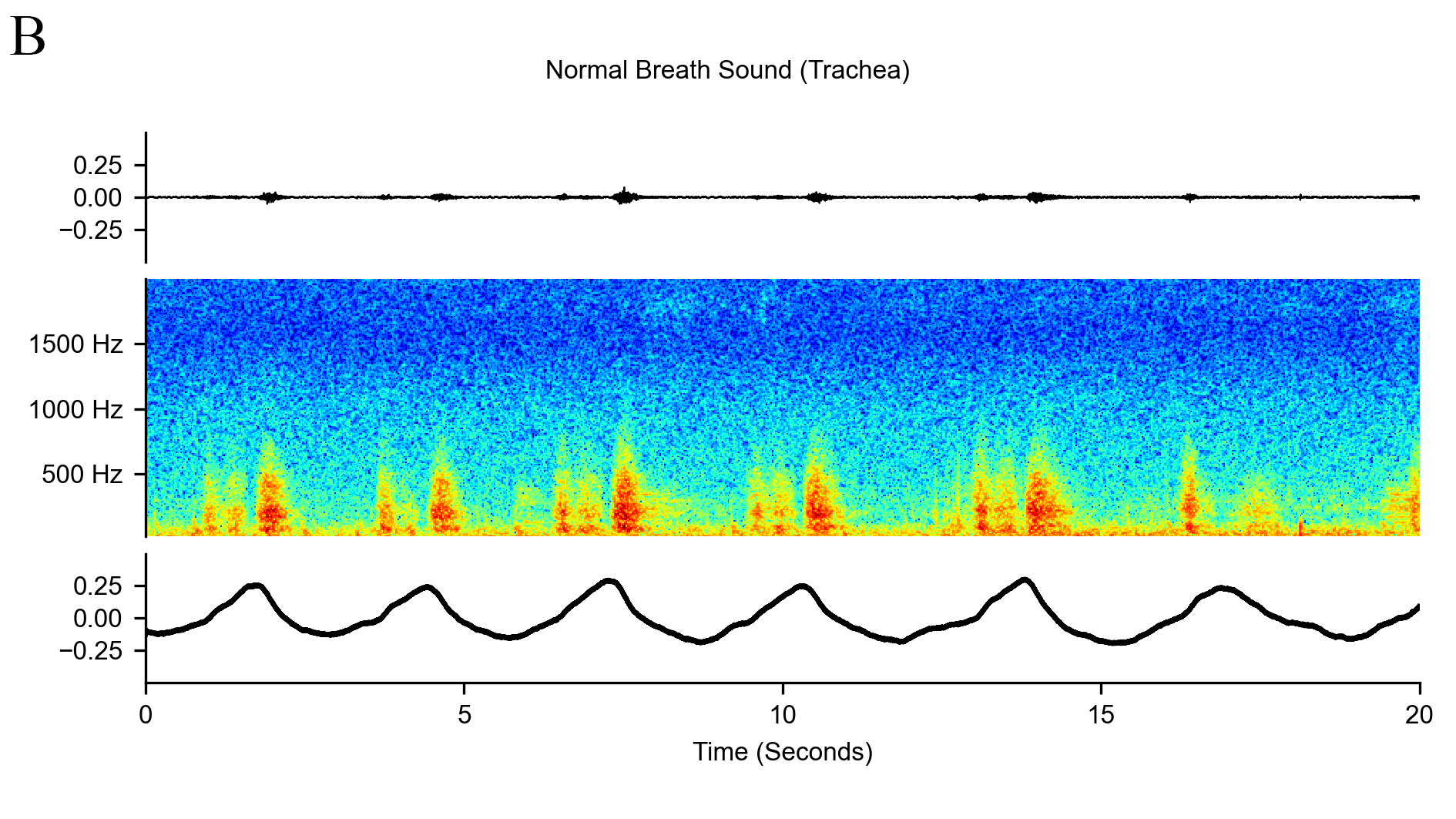

Supplement: Supplementary file 1 — Data S1. Supplementary Item 1. Visual representation of breath sounds as waveform (top) and spectrogram (middle), and plethysmography breathing pattern (bottom) where increasing values represent inspiration and decreasing values expiration. The x‐axis represents time in seconds. In the waveform, the y‐axis represents the amplitude or “loudness.” In the spectrogram, the y‐axis represents frequency in Hertz, and the intensity of sounds is represented as nuance color. (A) Four normal respiratory cycles over the lungs, (B) 6 normal respiratory cycles over the trachea, (C) 5 respiratory cycles over the lungs containing expiratory wheezes (thin transverse bands at 700 Hz), (D) 14 respiratory cycles over the lungs containing both wheezes (500 Hz) and crackles (high‐frequency and high‐intensity peaks), (E) 4 respiratory cycles over the lungs of increased intensity containing crackles, (F) 5 respiratory cycles over the trachea containing rattles (high‐frequency and high‐intensity peaks) and expiratory wheezes, (G) series of 3 coughs over the lungs containing wheezes, as well as an isolated wheeze between the second and third cough. The audio files corresponding to each figure are appended in the supplementary items. [file JVIM-38-495-s002.zip › Supplemetary figure 1B_Normal Breath Sound (Trachea).tif]

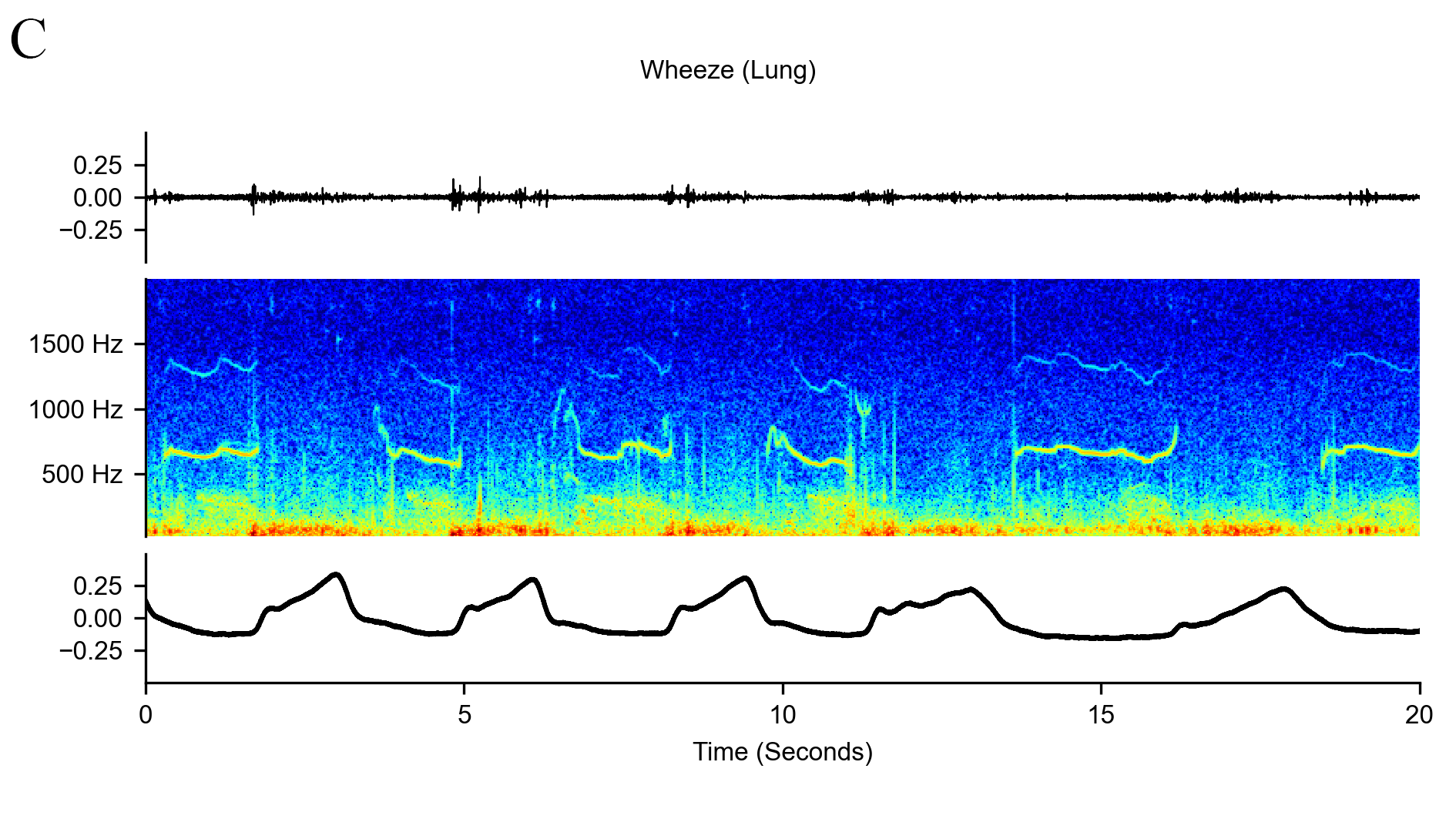

Supplement: Supplementary file 1 — Data S1. Supplementary Item 1. Visual representation of breath sounds as waveform (top) and spectrogram (middle), and plethysmography breathing pattern (bottom) where increasing values represent inspiration and decreasing values expiration. The x‐axis represents time in seconds. In the waveform, the y‐axis represents the amplitude or “loudness.” In the spectrogram, the y‐axis represents frequency in Hertz, and the intensity of sounds is represented as nuance color. (A) Four normal respiratory cycles over the lungs, (B) 6 normal respiratory cycles over the trachea, (C) 5 respiratory cycles over the lungs containing expiratory wheezes (thin transverse bands at 700 Hz), (D) 14 respiratory cycles over the lungs containing both wheezes (500 Hz) and crackles (high‐frequency and high‐intensity peaks), (E) 4 respiratory cycles over the lungs of increased intensity containing crackles, (F) 5 respiratory cycles over the trachea containing rattles (high‐frequency and high‐intensity peaks) and expiratory wheezes, (G) series of 3 coughs over the lungs containing wheezes, as well as an isolated wheeze between the second and third cough. The audio files corresponding to each figure are appended in the supplementary items. [file JVIM-38-495-s002.zip › Supplemetary figure 1C_Wheeze (Lung).tif]

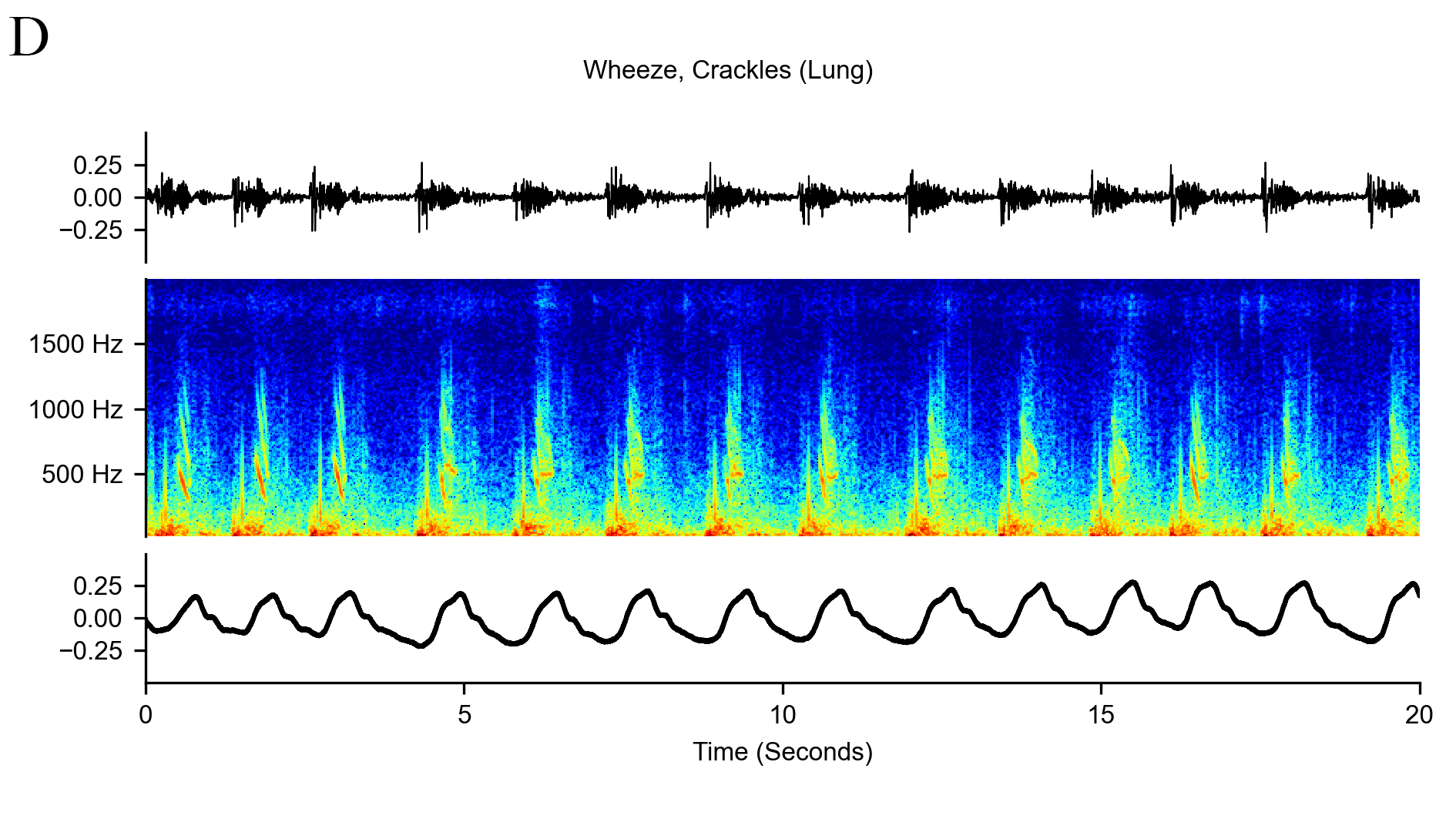

Supplement: Supplementary file 1 — Data S1. Supplementary Item 1. Visual representation of breath sounds as waveform (top) and spectrogram (middle), and plethysmography breathing pattern (bottom) where increasing values represent inspiration and decreasing values expiration. The x‐axis represents time in seconds. In the waveform, the y‐axis represents the amplitude or “loudness.” In the spectrogram, the y‐axis represents frequency in Hertz, and the intensity of sounds is represented as nuance color. (A) Four normal respiratory cycles over the lungs, (B) 6 normal respiratory cycles over the trachea, (C) 5 respiratory cycles over the lungs containing expiratory wheezes (thin transverse bands at 700 Hz), (D) 14 respiratory cycles over the lungs containing both wheezes (500 Hz) and crackles (high‐frequency and high‐intensity peaks), (E) 4 respiratory cycles over the lungs of increased intensity containing crackles, (F) 5 respiratory cycles over the trachea containing rattles (high‐frequency and high‐intensity peaks) and expiratory wheezes, (G) series of 3 coughs over the lungs containing wheezes, as well as an isolated wheeze between the second and third cough. The audio files corresponding to each figure are appended in the supplementary items. [file JVIM-38-495-s002.zip › Supplemetary figure 1D_Wheeze Crackles (Lung).tif]

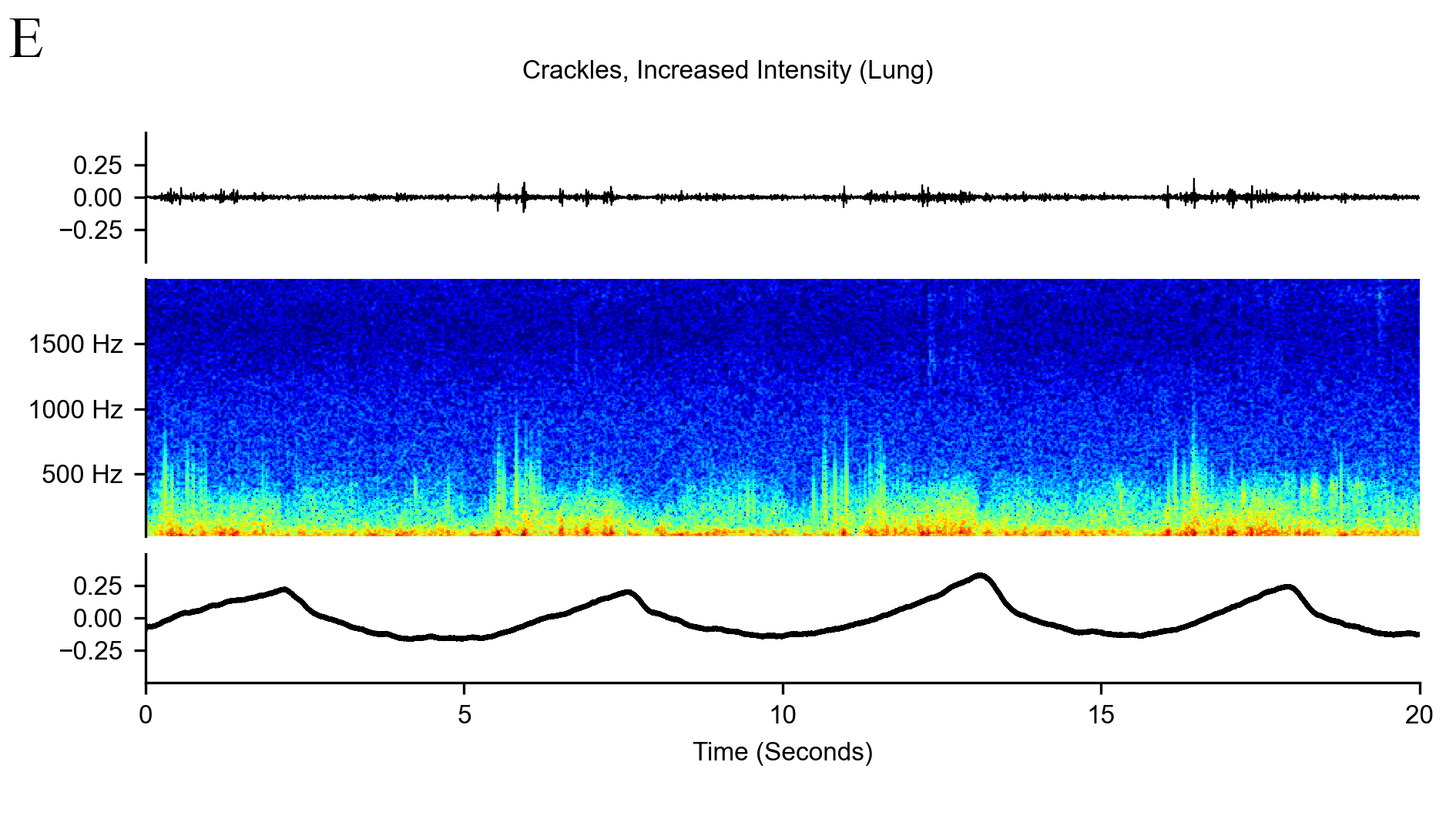

Supplement: Supplementary file 1 — Data S1. Supplementary Item 1. Visual representation of breath sounds as waveform (top) and spectrogram (middle), and plethysmography breathing pattern (bottom) where increasing values represent inspiration and decreasing values expiration. The x‐axis represents time in seconds. In the waveform, the y‐axis represents the amplitude or “loudness.” In the spectrogram, the y‐axis represents frequency in Hertz, and the intensity of sounds is represented as nuance color. (A) Four normal respiratory cycles over the lungs, (B) 6 normal respiratory cycles over the trachea, (C) 5 respiratory cycles over the lungs containing expiratory wheezes (thin transverse bands at 700 Hz), (D) 14 respiratory cycles over the lungs containing both wheezes (500 Hz) and crackles (high‐frequency and high‐intensity peaks), (E) 4 respiratory cycles over the lungs of increased intensity containing crackles, (F) 5 respiratory cycles over the trachea containing rattles (high‐frequency and high‐intensity peaks) and expiratory wheezes, (G) series of 3 coughs over the lungs containing wheezes, as well as an isolated wheeze between the second and third cough. The audio files corresponding to each figure are appended in the supplementary items. [file JVIM-38-495-s002.zip › Supplemetary figure 1E_Crackles Increased Intensity (Lung).tif]

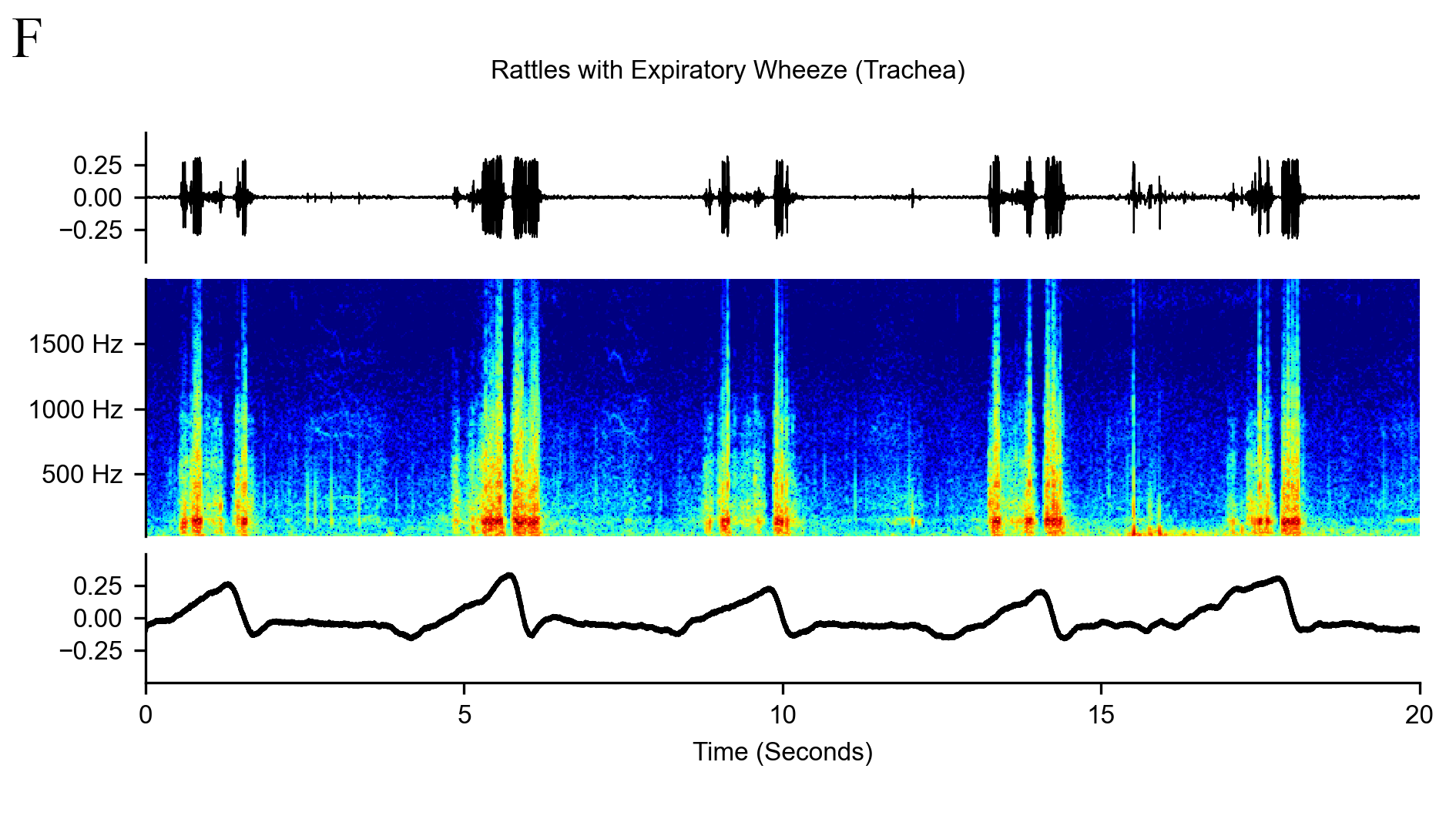

Supplement: Supplementary file 1 — Data S1. Supplementary Item 1. Visual representation of breath sounds as waveform (top) and spectrogram (middle), and plethysmography breathing pattern (bottom) where increasing values represent inspiration and decreasing values expiration. The x‐axis represents time in seconds. In the waveform, the y‐axis represents the amplitude or “loudness.” In the spectrogram, the y‐axis represents frequency in Hertz, and the intensity of sounds is represented as nuance color. (A) Four normal respiratory cycles over the lungs, (B) 6 normal respiratory cycles over the trachea, (C) 5 respiratory cycles over the lungs containing expiratory wheezes (thin transverse bands at 700 Hz), (D) 14 respiratory cycles over the lungs containing both wheezes (500 Hz) and crackles (high‐frequency and high‐intensity peaks), (E) 4 respiratory cycles over the lungs of increased intensity containing crackles, (F) 5 respiratory cycles over the trachea containing rattles (high‐frequency and high‐intensity peaks) and expiratory wheezes, (G) series of 3 coughs over the lungs containing wheezes, as well as an isolated wheeze between the second and third cough. The audio files corresponding to each figure are appended in the supplementary items. [file JVIM-38-495-s002.zip › Supplemetary figure 1F_Rattles with Expiratory Wheeze (Trachea).tif]

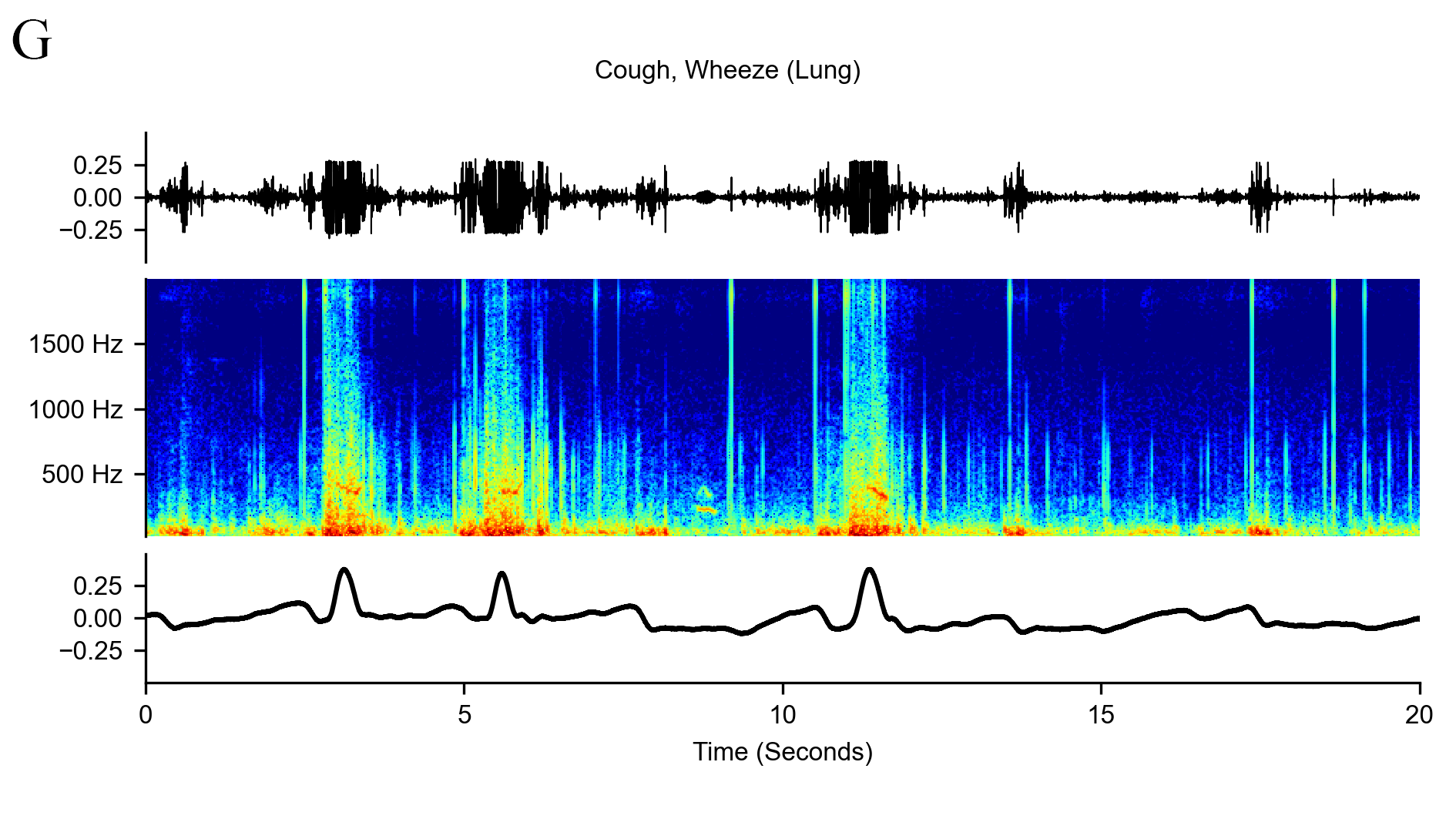

Supplement: Supplementary file 1 — Data S1. Supplementary Item 1. Visual representation of breath sounds as waveform (top) and spectrogram (middle), and plethysmography breathing pattern (bottom) where increasing values represent inspiration and decreasing values expiration. The x‐axis represents time in seconds. In the waveform, the y‐axis represents the amplitude or “loudness.” In the spectrogram, the y‐axis represents frequency in Hertz, and the intensity of sounds is represented as nuance color. (A) Four normal respiratory cycles over the lungs, (B) 6 normal respiratory cycles over the trachea, (C) 5 respiratory cycles over the lungs containing expiratory wheezes (thin transverse bands at 700 Hz), (D) 14 respiratory cycles over the lungs containing both wheezes (500 Hz) and crackles (high‐frequency and high‐intensity peaks), (E) 4 respiratory cycles over the lungs of increased intensity containing crackles, (F) 5 respiratory cycles over the trachea containing rattles (high‐frequency and high‐intensity peaks) and expiratory wheezes, (G) series of 3 coughs over the lungs containing wheezes, as well as an isolated wheeze between the second and third cough. The audio files corresponding to each figure are appended in the supplementary items. [file JVIM-38-495-s002.zip › Supplemetary figure 1G_Cough Wheeze (Lung).tif]
