# Supplementary material for: Breath characteristics and adventitious lung sounds in healthy and asthmatic horses
Source: J Vet Intern Med. 2024 Jan 8;38(1):495–504. doi: 10.1111/jvim.16980 (PMC10800186; doi:10.1111/jvim.16980)
Supplement: Supplementary file 2 — Data S2. Supplementary Item 2. Supplementary figure and table. [file JVIM-38-495-s001.pdf]

Supplementary Item 2

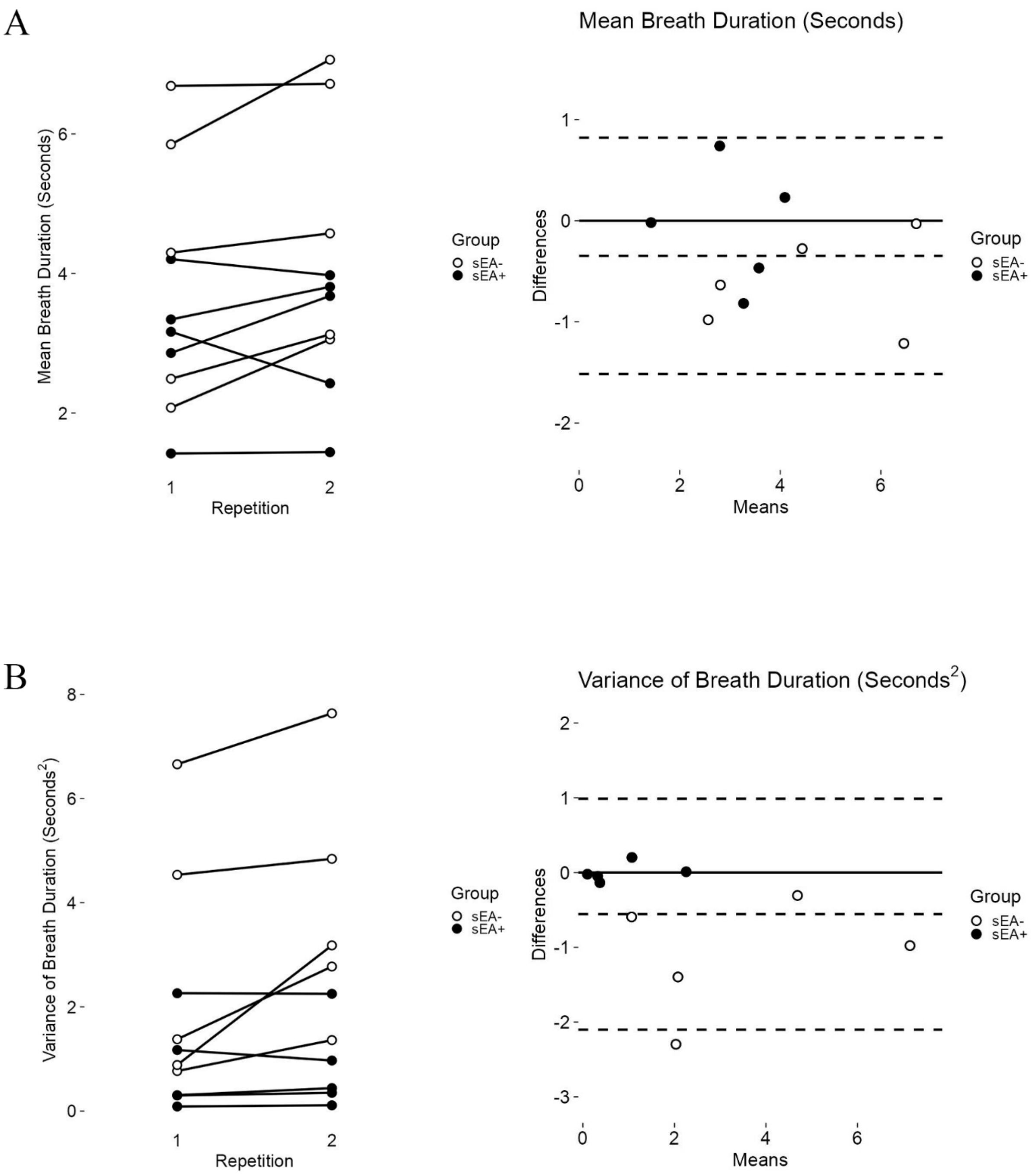

C

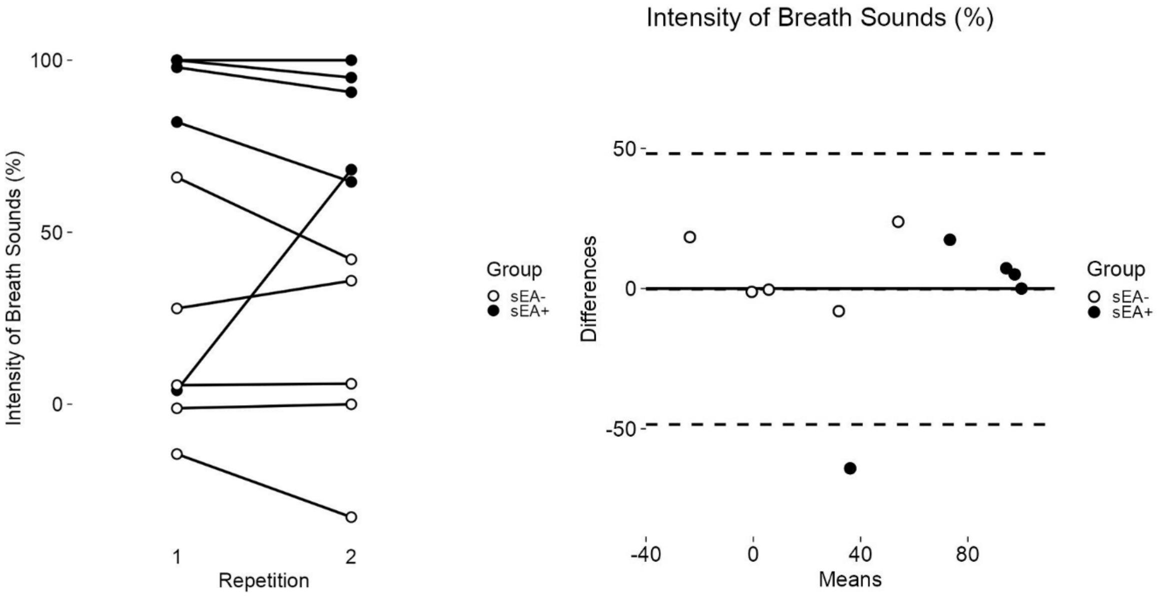

Intensity of Breath Sounds (%)
100
50
0
Intensity of Breath Sounds (%)
1
2
Repetition
Group
sEA-
sEA+
50
0
-50
Differences
-40
0
40
80
Means
Group
sEA-
sEA+

D

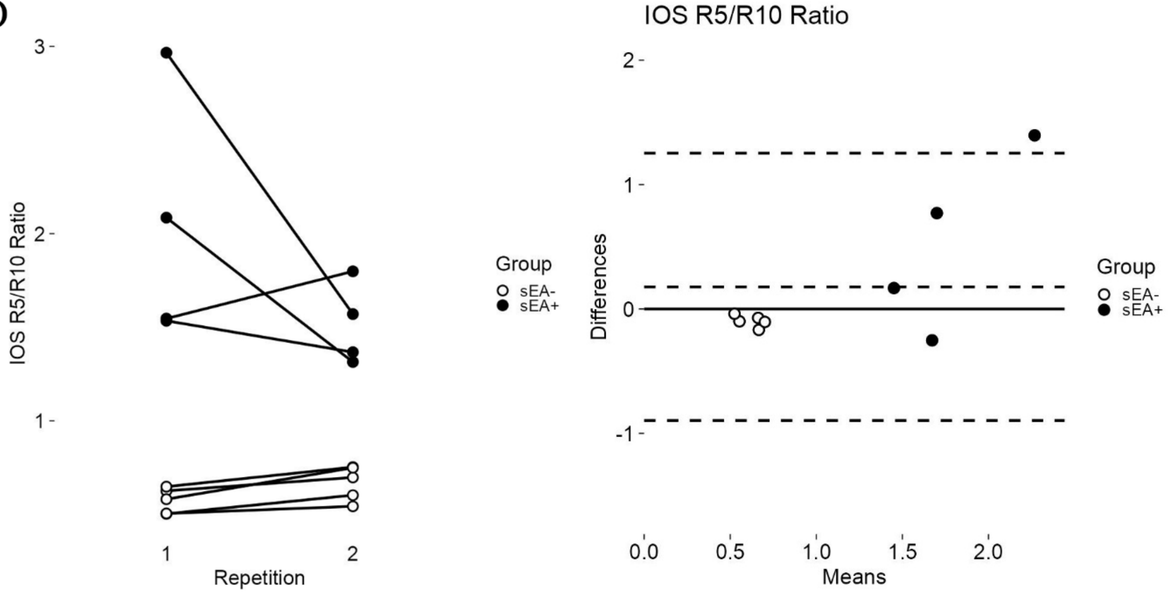

IOS R5/R10 Ratio
3
2
1
IOS R5/R10 Ratio
1
2
Repetition
Group
sEA-
sEA+
2
1
0
-1
Differences
0.0
0.5
1.0
1.5
2.0
Means
Group
sEA-
sEA+

E

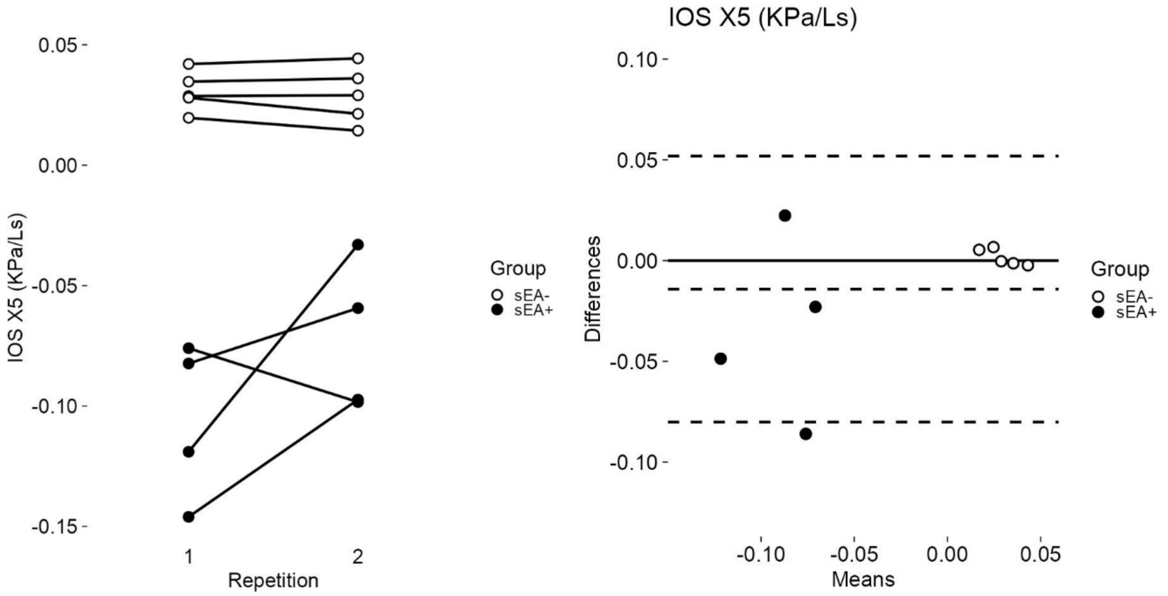

IOS X5 (KPa/Ls)
IOS X5 (KPa/Ls)
Repetition
Group
sEA-
sEA+
Differences
Means

F

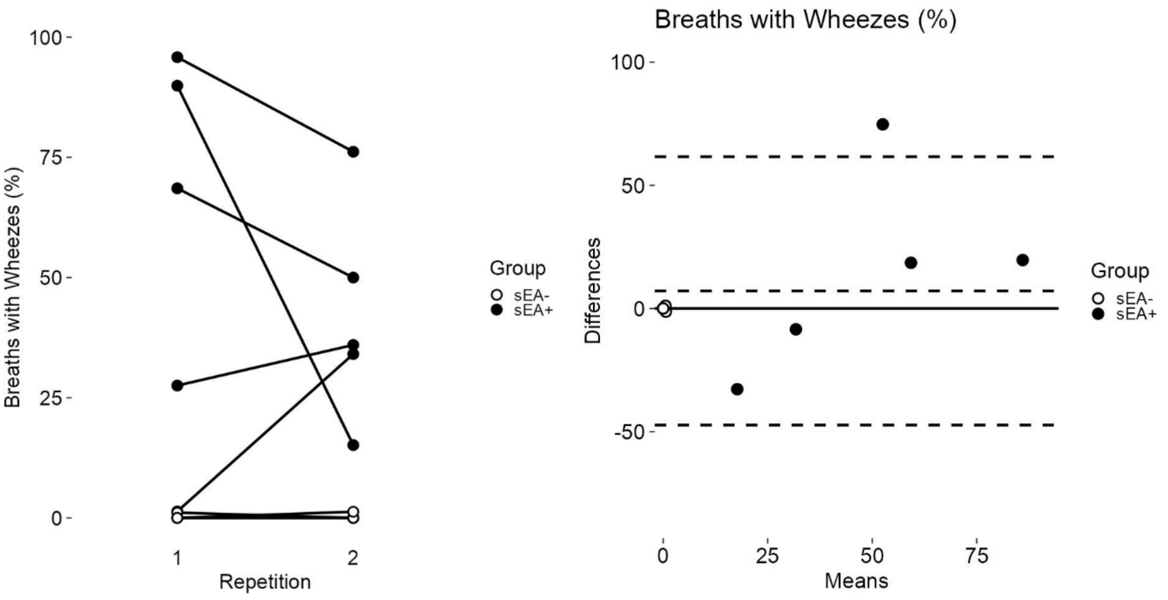

Breaths with Wheezes (%)
Breaths with Wheezes (%)
Repetition
Group
sEA-
sEA+
Differences
Means

G

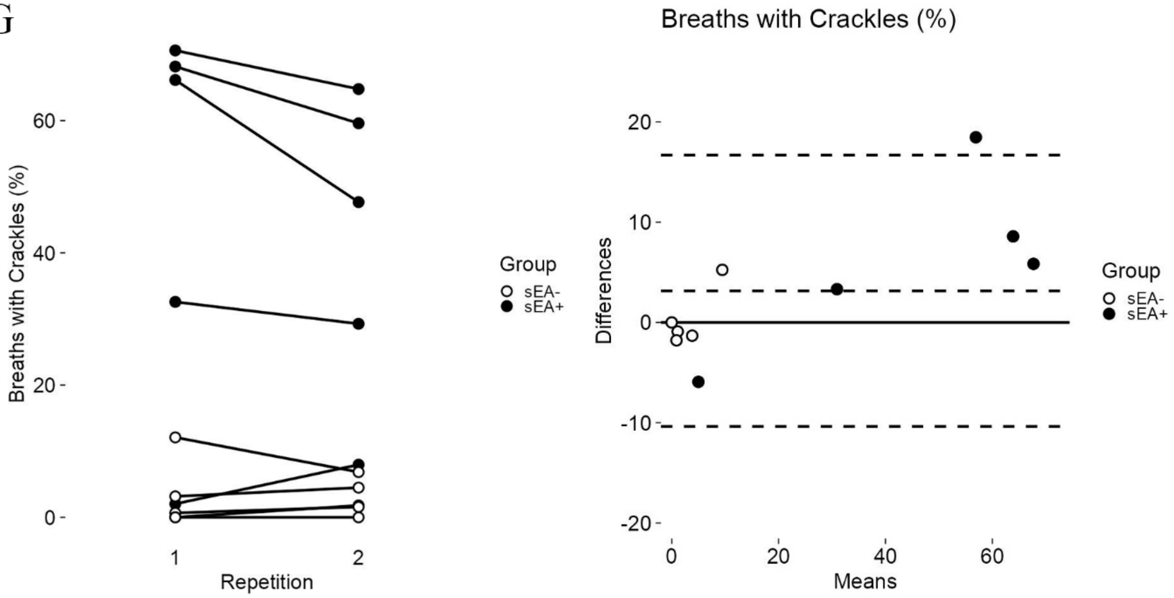

Breaths with Crackles (%)
Breaths with Crackles (%)
Repetition
Group
sEA-
sEA+
Differences
Means
Group
sEA-
sEA+

H

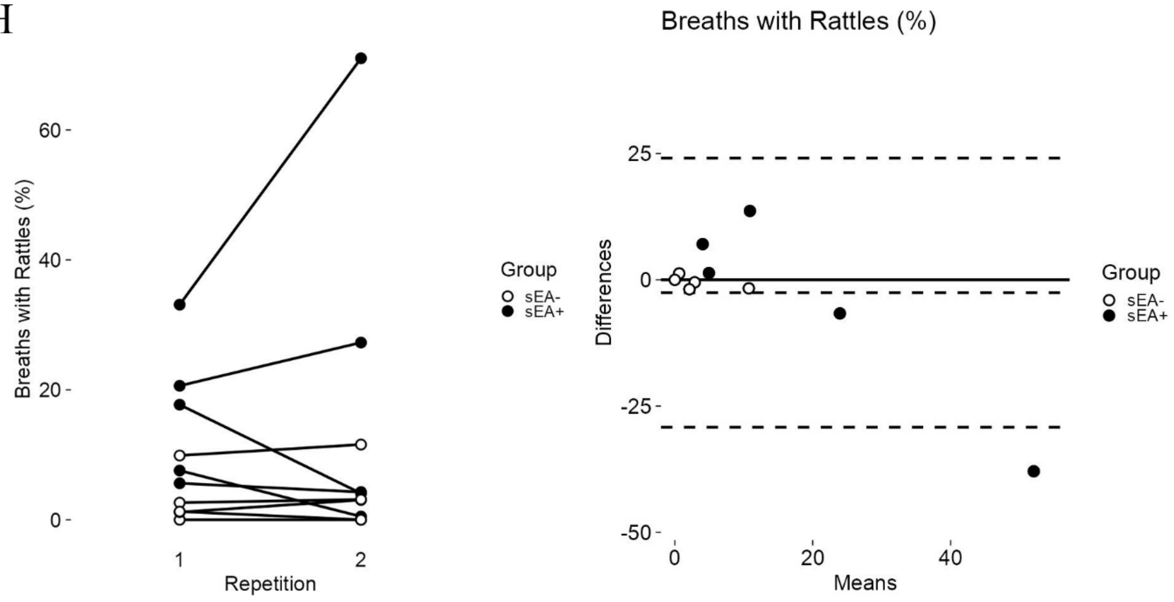

Breaths with Rattles (%)
Breaths with Rattles (%)
Repetition
Group
sEA-
sEA+
Differences
Means
Group
sEA-
sEA+

I

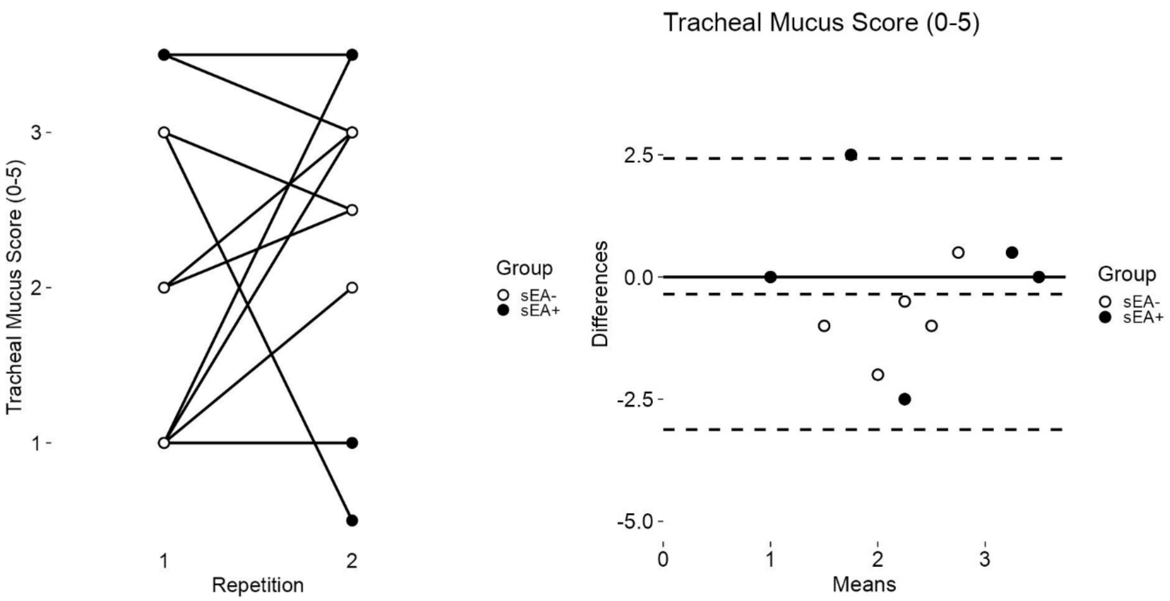
Tracheal Mucus Score (0-5)
Tracheal Mucus Score (0-5)
3
2
1
1
2
Repetition
Group
sEA-
sEA+
Differences
2.5
0.0
-2.5
-5.0
0
1
2
3
Means
Group
sEA-
sEA+

J

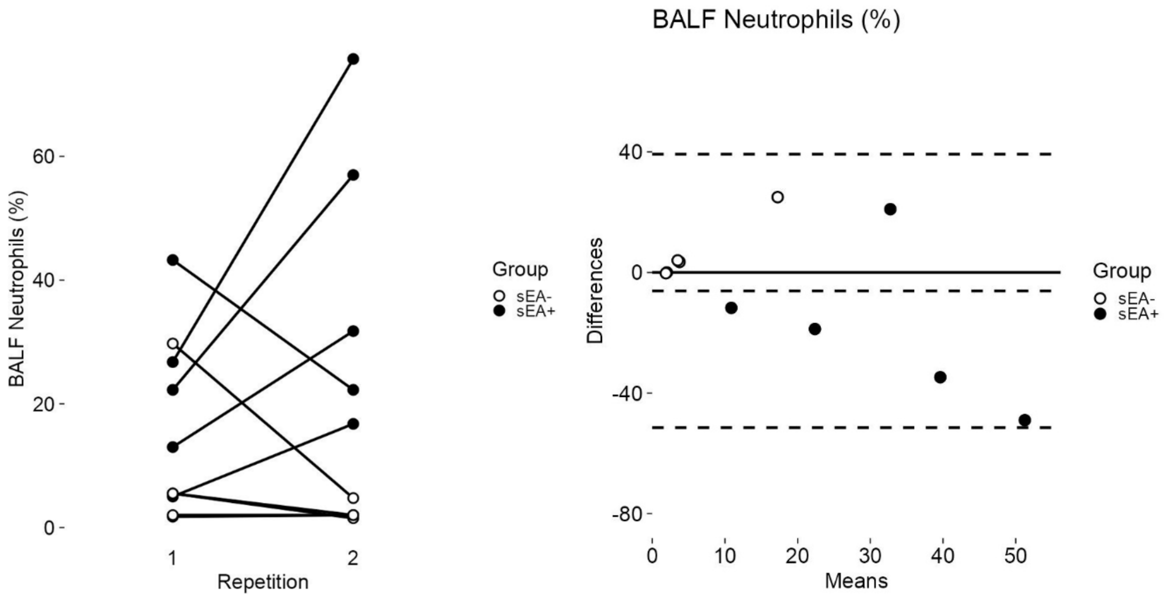
BALF Neutrophils (%)
BALF Neutrophils (%)
60
40
20
0
1
2
Repetition
Group
sEA-
sEA+
Differences
40
0
-40
-80
0
10
20
30
40
50
Means
Group
sEA-
sEA+

K

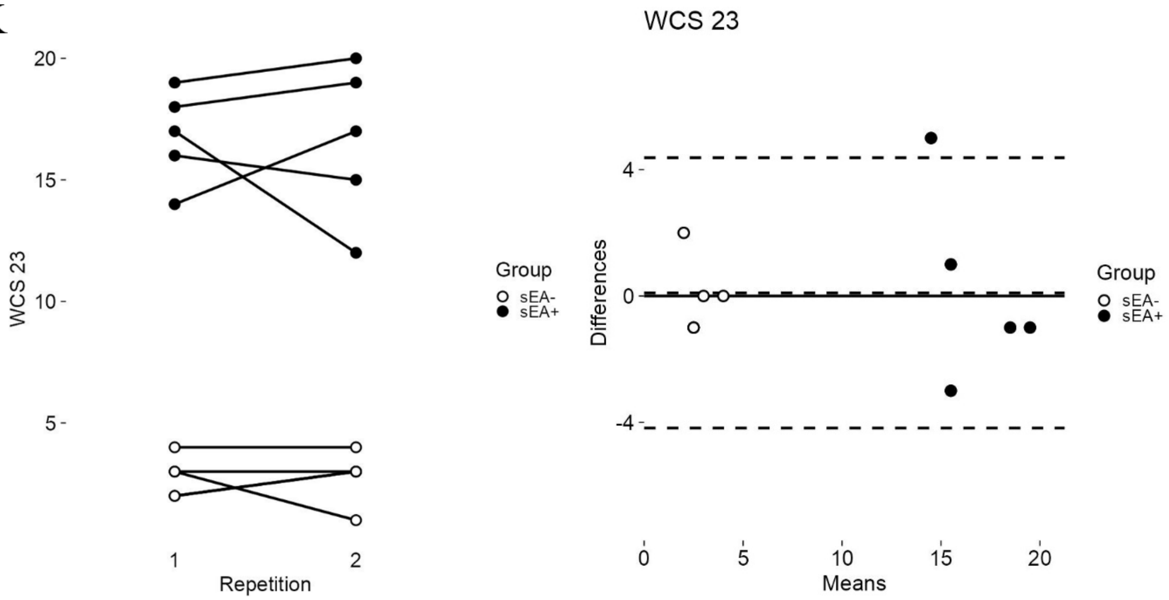

Supplementary figure: Spaghetti plots (left) and Bland-Altman plots (right) of repeated measurements in severely asthmatic horses in remission (sEA-) and exacerbation (sEA+) for means (A) and variance (B) of respiratory duration, breath intensity (C), ratio of resistance at 5 and 10 Hz during impulse oscillometry (IOS R5/R10) (D), reactance at 5 Hz during impulse oscillometry (IOS X5) (E), proportion of wheezes (F), crackles (G), and rattles (H), tracheal mucus score (I), neutrophilic percentage in bronchoalveolar lavage fluid (BALF) cytology (J), and 23-point weighted clinical score (WCS23) (K). One sEA+ Horse did not tolerate the oscillometry measurements, hence there are two missing values in the corresponding plots.

Supplementary table: t-test results for repeated measurements in horses with severe equine asthma (sEA).

	t	df	P	Mean	Standard Deviation
Mean of breath duration	1.840	9	1.00	0.347	0.596
Variance of breath duration	2.232	9	0.63	0.556	0.788
Intensity	0.025	9	1.00	0.192	24.636
IOS R5/R10	-0.972	8	1.00	-0.178	0.548
IOS X5	1.260	8	1.00	0.014	0.034
Proportion of wheezes	-0.815	9	1.00	-7.161	27.793
Proportion of crackles	-1.444	9	1.00	-3.152	6.905
Proportion of rattles	0.588	9	1.00	2.530	13.597
TMA	0.782	9	1.00	0.350	1.415
Neutrophilic percentage of BALF cytology	0.834	9	1.00	6.100	23.143
WCS23	-0.145	9	1.00	-0.100	2.183

Abbreviations: BALF, bronchoalveolar lavage fluid; IOS R5/R10, ratio of resistance at 5 and 10 Hz during impulse oscillometry; IOS X5, reactance at 5 Hz during impulse oscillometry; TMA, tracheal mucus accumulation; WCS23, 23-point weighted clinical score.
